# PbrWRKY62-PbrADC1 module involves in superficial scald development of *Pyrus bretschneideri* Rehd.fruit via regulating putrescine biosynthesis

**DOI:** 10.1186/s43897-024-00081-8

**Published:** 2024-02-20

**Authors:** Xu Zhang, Lijuan Zhu, Ming Qian, Li Jiang, Peng Gu, Luting Jia, Chunlu Qian, Weiqi Luo, Min Ma, Zhangfei Wu, Xin Qiao, Libin Wang, Shaoling Zhang

**Affiliations:** 1 State Key Laboratory of Crop Genetics and Germplasm Enhancement, Nanjing Agricultural University, Nanjing, 210095 Jiangsu China; 2 College of Food Science and Technology, Nanjing Agricultural University, Nanjing, 210095 Jiangsu China; 3 College of Food Science and Engineering, Yangzhou University, Yangzhou, 225127 Jiangsu China; 4 Center for Integrated Pest Management, North Carolina State University, Raleigh, NC 27606 USA; 5 Joint Center for Single Cell Biology, School of Agriculture and Biology, Shanghai Jiao Tong University, Shanghai, 200240 China

**Keywords:** *P. bretschneideri* Rehd., Superficial scald development, Putrescine biosynthesis, PbrWRKY62-PbrADC1 module, Gene expression, Metabolomics

## Abstract

Putrescine plays a role in superficial scald development during the cold storage of pear fruit. However, the molecular mechanism behind this phenomenon has not been un-fully clarified until recently. In this study, a conjoint analysis of metabolites and gene expression profiles in the putrescine-metabolic pathway of *P. bretschneideri* Rehd. fruit followed by experimental validation revealed that PbrADC1, forming a homodimer in the chloroplast, was involved in putrescine biosynthesis and thus fruit chilling resistance. Additionally, the substrate-binding residue Cys^546^ in PbrADC1, whose activity was modified by H_2_O_2_, played a crucial role in arginine decarboxylation into agmatine. Through a combined analysis of the distribution of *cis*-acting elements in the *PbrADC1* promoter as well as the expression profiles of related transcription factors (TFs), several TFs were identified as upstream regulators of *PbrADC1* gene. Further investigation revealed that the nuclear PbrWRKY62 could directly bind to the W-box elements in the *PbrADC1* promoter, activate its expression, enhance putrescine accumulation, and thus increase fruit chilling tolerance. In conclusion, our results suggest that the PbrWRKY62-PbrADC1 module is involved in the development of superficial scald in *P. bretschneideri* Rehd. fruit via regulating putrescine biosynthesis. Consequently, these findings could serve as valuable genetic resources for breeding scald-resistant pear fruit.

**Graphical Abstract:**

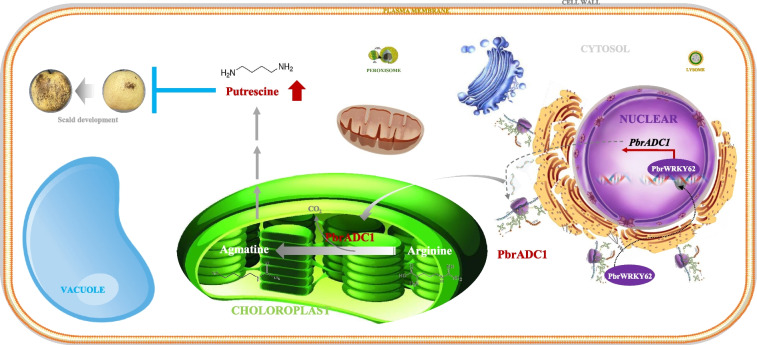

**Supplementary Information:**

The online version contains supplementary material available at 10.1186/s43897-024-00081-8.

## Core

The expression levels of *PbrWRKY62* and *PbrADC1* were upregulated by chilling exposure; and the nuclear PbrWRKY62 could directly bind to the W-box elements in *PbrADC1* promoter and then activate its expression, enhancing putrescine accumulation and thus chilling tolerance of fruit.

## Gene and accession numbers

Sequence data from this article can be found in the database of the pear genome database (http://peargenome.njau.edu.cn/) under the accession numbers: *PbrADC1* (Pbr022368.1), and *PbrWRKY62* (Pbr001424.1).

## Introduction

Low-temperature storage, which is commonly applied to prolong the storability of apple and pear fruit, could lead to the development of superficial scald, demonstrating as brown or black patches on the fruit epidermis (Giné-Bordonaba et al. 2020; Qian et al. 2021). Superficial scald is considered a chilling injury symptom (Watkins et al. 1995) and is believed to be associated with the disruption of the cellular redox homeostasis (Qian et al. 2021). Chilling exposure can impair the cytochrome pathway of electron transport, leading to the accumulation of superoxide free radicals (O_2_^•−^) and hydrogen peroxide (H_2_O_2_) (Gong et al. 2021; Hui et al. 2016). When these two compounds interact, hydroxyl radicals (OH^•^) are formed for the oxidation of α-farnesene, ultimately resulting in the formation of conjugated trienols (CTols) and 6-methyl-5-hepten-2-one (MHO) (Gong et al. 2021; Hui et al. 2016). MHO is the direct trigger of superficial scald (Hui et al. 2016). During the cold storage of pear fruit, reactive oxygen species (ROS) and CTols gradually accumulate, with a MHO burst when superficial scald symptom occurs (Feng et al. 2018; Hui et al. 2016). Furthermore, the fumigation of pear fruit with exogenous MHO has been shown to promote superficial scald development (Hui et al. 2016).

Over the past half-century, various physicochemical treatments have been explored to control the development of superficial scald. These treatments include 1-MCP fumigation, diphenylamine (DPA) dipping, resveratrol immersion, modified atmosphere packaging (MAP), and controlled atmosphere (CA) storage (Dias et al. 2020; Feng et al. 2018; Li et al. 2016; Mditshwa et al. 2017; Poirier et al. 2020; Qian et al. 2021). The effectiveness of these postharvest handling practices in mitigating superficial scald development may be attributed to their ability to enhance ROS scavenging capacity in fruit, thereby inhibiting the production of MHO (Feng et al. 2018; Hui et al. 2016; Qian et al. 2021). For instance, 1-MCP fumigation could upregulate the activities of superoxide dismutase (SOD) and catalase (CAT), reduce the production rate of O_2_^•−^, decrease CTols content, delay MHO burst, and consequently suppress superficial scald development in pear fruit (Feng et al. 2018; Hui et al. 2016).

As low-molecular-weight and aliphatic polycations, polyamines participate in plant abiotic stress responses via binding to anionic macromolecules, regulating transcription and translation, and modulating the antioxidant system (Liu et al. 2015; Winter et al. 2015). The most common polyamines in plants include putrescine, spermidine, and spermine (Xiang et al. 2022). Chilling exposure stimulates the accumulation of endogenous polyamines in the epidermal tissue of pear fruit; moreover, the application of exogenous putrescine can serve to mitigate superficial scald development (Calvo et al. 2018; Li et al. 2021a). These results collectively suggest that putrescine play a role in superficial scald development. Further studies have illustrated that the putrescine-mediated inhibition of superficial scald development in pear fruit is associated with the maintenance of mitochondrial number and morphological integrity, the improvement of mitochondrial function, the suppression of the increase or amplitude of mitochondrial membrane permeability transition pore (MPTP) opening, and the reduction of mitochondrial ROS accumulation (Li et al. 2021a).

In plants, putrescine is synthesized either through the decarboxylation of ornithine by ornithine decarboxylase (ODC) or through the arginine decarboxylase (ADC) pathway, which involves three enzymes: ADC, agmatine iminohydrolase (AIH), and *N*-carbamoylputrescine amidase (NLP) (Fig. S1) (Kou et al. 2018). Upon formation, putrescine can be further converted into spermidine and spermine by spermidine synthase (SPDS) and spermine synthase (SPMS) (Fig. S1) (Winter et al. 2015). Alternatively, ornithine and arginine can be interconverted with the assistance of ornithine transcarbamylase (OTC), arginosuccinate synthase (ASS), arginosuccinate lyase (ASL), nitric oxide synthase-like (NOS), and arginase (ARG) (Fig. S1) (Winter et al. 2015).

With the rapid progress in sequencing technologies, various plant genomes have been explored, thus facilitating the alternation of fruit traits by manipulating the expression of a specific gene (Chagné et al. 2014; Dong et al. 2020; Wu et al. 2013). Heterogenous overexpression of an oat *ADC* promoted putrescine biosynthesis in rice (Capell et al. 1998). Similarly, homogenous overexpression of *SaADC1*, whose mRNA level demonstrated a positive association with putrescine content during the cold storage of the freezing-tolerant potato, would enhance putrescine accumulation and, consequently, the freezing tolerance of transgenic plant (Kou et al. 2018). In addition to *ADC*, other genes in the putrescine-metabolic pathway play roles in plant responses to abiotic stresses as well. Knockout of *Arabidopsis AtARG1/2* elevated putrescine level but suppressed ROS accumulation, enhancing plant tolerance to water deficit, salt, and freezing stresses, while the opposite phenomenon was observed in the *AtARG1/2*-overexpressing lines (Shi et al. 2013).

In plants, the transcription of structural genes is controlled by transcript factors (TFs) through their interaction with the corresponding *cis*-acting elements in the gene promoters (Chakravarthy et al. 2003; Liu et al. 2015; Wu et al. 2016). Common TF families, such as WRKYs, MYBs, bZIPs, NACs, and CBFs, play a crucial role in plant adaption to abiotic stresses (Liu et al. 2015; Song et al. 2022). For example, CsCBF1 can bind to the *CsADC* promoter and activate its expression, leading to the elevated putrescine level and the enhanced chilling resistance of *Citrus sinensis* (Song et al. 2022). Similarly, FcWRKY70 has been explored to improve the drought tolerance of *Fortunella crassifolia* via modulating *FcADC* expression and then putrescine production (Gong et al. 2015). In contrast, the nuclear PtrNAC72 from *Poncirus trifoliata* acts as a transcriptional repressor of *PtADC*, compromising putrescine formation and, consequently, plant drought tolerance (Wu et al. 2016). However, our understanding on the alternation of polyamine metabolism during superficial scald development in pear fruit as well as the regulatory mechanism remains limited.

In this study, we characterized a chloroplast-located PbrADC1 and its upstream regulator, PbrWRKY62, from *P. bretschneideri* Rehd. genome. We validated their involvement in superficial scald development by regulating putrescine biosynthesis. Furthermore, the substrate-binding residue (Cys^546^) of PbrADC1 and the impact of exogenous H_2_O_2_ treatment on its activity were also explored.

## Result

### Identification of PbrADC1 as the candidate gene involved in the development of superficial scald in pear

**Fig. 1:**
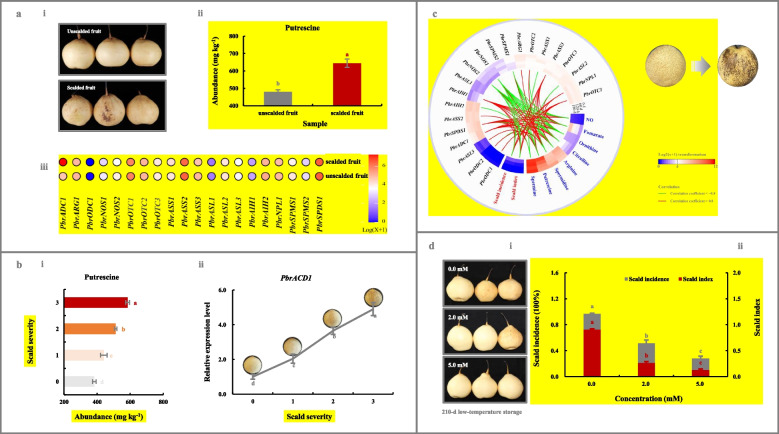
Characterization of *PbrADC1* gene involved in superficial scald development via regulating putrescine biosynthesis. **a** Alternation of putrescine metabolism upon superficial scald development in ‘Yali’ fruit. (a-i) Visual quality change. (a-ii) Putrescine level. (a-iii) Expression profiles of 21 putrescine-metabolism-related genes. ‘Yali’ fruit, with and without superficial scald, were sampled after -0.5 ℃ for 180 d followed by a 7-d shelf life at 20 ℃. **b** Putrescine content and *PbrADC1* expression abundance in ‘Yali’ fruit of different superficial scald severities. (b-i) Putrescine content. (b-ii) *PbrADC1* mRNA abundance. ‘Yali’ fruit, with different superficial scald severities (Hui et al. 2016), were sampled after -0.5 ℃ for 180 d followed by a 7-d shelf life at 20 ℃. The expression abundance of *PbrADC1* in the unscalded fruit (Severity 0) was set as 1.0 for RT-qPCR assay. **c** Dynamic change of putrescine metabolism during cold storage of ‘Dangshansuli’ fruit. ‘Dangshansuli’ fruit were sampled every 60-d storage at 0.5 ℃ followed by a 7-d shelf life at 25 ℃. **d** Impact of exogenous putrescine treatment on superficial scald development in pear fruit. (d-i) Visual quality change. (d-ii) Superficial scald incidence and index. ‘Yali’ fruit were randomly divided into three groups for different treatments: 0.0 (H_2_O), 2.0-mM, and 5.0-mM putrescine immersion for 15 min before 210-d storage at -0.5 ℃ followed by a 7-d shelf life at 20 ℃. A total of 21 putrescine-metabolism-related genes were characterized from the *P. bretschneideri* Rehd. genome (Table S2). Data represent the mean values of three biological replicates, except for the transcriptome assay of the (un) scalded ‘Yali’ fruit (two replicate). Different lowercase letters meant significance between samples (*p* < 0.05). Color scale represented normalized log2-transformed (FPKM + 1), where red indicated a high level, blue represented a low level, and white indicated a medium level. Absolute correlation coefficients between attributes ≥ 0.8 were visualized in the heatmap, where red lines demonstrated extremely strong positive correlations, while green demonstrated extremely strong negative associations

In order to assay the relationship between polyamine metabolism and superficial scald development in pear fruit, several experiments were carried out. As illustrated in Fig. 1a-i/ii, a higher level of putrescine was observed in the scalded ‘Yali’ fruit when compared with that in the unscaled fruit, and its contents demonstrated a positive association with superficial scald severities (Fig. 1b-i). In a further study using ‘Dangshansuli’ fruit as the material, superficial scald incidence and index increased during cold storage, accompanied by the accumulation of putrescine, arginine, ornithine, citrulline, NO, and fumarate, but a decrease in spermidine (Fig. 1c). Additionally, the application of exogenous putrescine inhibited superficial scald development, and the effect was correlated with concentration (Fig. 1d). These results suggested that putrescine played a role in superficial scald development.

To explore the candidate genes responsible for the alternation of putrescine metabolism during scald development, we firstly identified the members in putrescine-metabolic pathway. As shown in Table S2, 19 putrescine-metabolism-related genes were characterized from pear by the BLASTP searches of the *P. bretschneideri* Rehd. genome, while two *ODC* genes were identified based on transcriptome annotation. They were distributed on 10 chromosomes with uneven non-random distributions (Fig. S2a and Table S2). The physico-chemical characteristics of the corresponding proteins were summarized in Table S2. Most (15) of them originated from whole-genome duplication (WGD)/segmental duplications (Table S3). Consistent with this, highly conserved synteny was identified in the regions containing these genes (Fig. S2b), with Ka/Ks ratios of all paralogous genes < 1.0 (Table S4). Further analysis revealed that exon numbers in their genomic sequences were diverse (Fig. S2c-ii), and a total of 91 *cis*-acting regulatory elements were identified from their promoters (Fig. S2c-iii).

Except for *PbrODC2*, 20 putrescine-metabolism-related genes were transcribed in ‘Yali’ fruit after 180-d chilling storage (Fig. 1a-iii and Table S5). Notably, only the expression level of *PbrADC1* was upregulated upon superficial scald development (fold change ≥ 2.0 and FDR < 0.05, Fig. 1a-iii and Table S5), and it demonstrated positive correlations with putrescine level and superficial scald severity (Fig. 1b). Similar results were observed during cold storage of ‘Dangshansuli’ fruit: out of 21 transcribed members, the mRNA abundances of *PbrADC1*, *PbrAIH1*, and *PbrSPDS1* gradually increased, and displayed extremely strong positive associations with putrescine level and superficial scald incidence/index (correlation coefficient ≥ 0.8; Fig. 1c and Table S6); on the other hand, an opposite phenomenon was observed for *PbrNOS2* and *PbrSPMS1* (Fig. 1c and Table S6). RT-qPCR assay validated the transcriptome result on the expression pattern of *PbrADC1* during low-temperature storage of ‘Dangshansuli’ fruit (Fig. 1c & S3a).

In combination with Liu et al. (2015) and Kou et al. (2018)’s reports, *PbrADC1* might be involved in superficial scald development via regulating putrescine biosynthesis and thus was selected for further analysis. Further study found that the CDS sequences and the corresponding protein sequences of *PbrADC1* in ‘Dangshansuli’ and ‘Yali’ fruits were highly identical (Fig. S4a/b-i).

### Functional validation of PbrADC1 involved in putrescine biosynthesis and thus chilling resistance

**Fig. 2:**
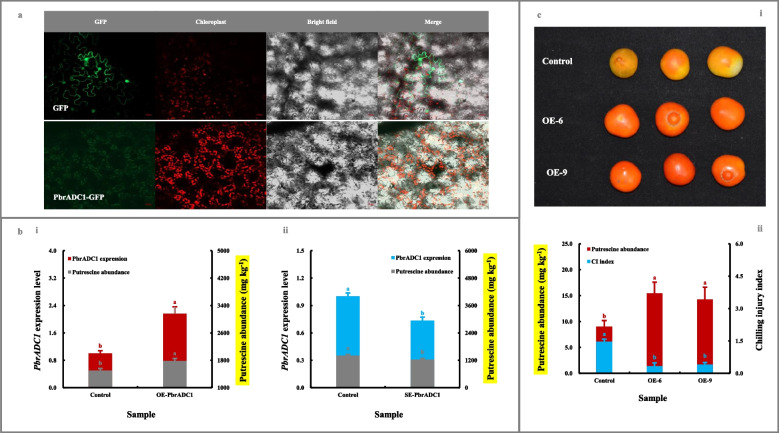
Functional validation of PbrADC1 involved in fruit putrescine biosynthesis and thus chilling resistance. **a** Subcellular localization of PbrADC1. The recombinant pBI221-*PbrADC1* vector was transformed into *N. benthamiana* leaves before fluorescence signal detection. **b** Impact of transient genetic transformation in the ripe ‘Dangshansuli’ fruit on putrescine abundance. (b-i) Transient overexpression of *PbrADC1* gene. The ripe ‘Dangshansuli’ fruit transformed with the empty vector was used as a control. (b-ii) Transient silence of *PbrADC1* gene. The ripe ‘Dangshansuli’ fruit co-transformed with empty pTRV2 and pTRV1 was used as a control. The expression abundance of *PbrADC1* in control fruit was set as 1.0 for RT-qPCR assay. **c** Impact of overexpressing *PbrADC1* gene in tomato on fruit putrescine biosynthesis and chilling resistance. (c-i) Visual quality change. (c-ii) Putrescine level and chilling injury index. Tomato fruits at 35 DAFB, including the wide-type (control) and the *PbrADC1*-overexpressing (OE) lines, were harvested and then exposed to 4 ℃ for 10 d followed by 20 ℃ storage for 7 d. Data represented the mean value of three biological replicates, and different lowercase letters meant significance between samples (*p* < 0.05)

As shown in Fig. 2a, PbrADC1, without any signal peptide and transmembrane domain (Fig. S5a/b-i), accumulated in the chloroplasts of *N. benthamiana* leaves.

To validate the role of PbrADC1 in putrescine biosynthesis, we first assay its function in pear fruit. When compared with the control (empty vector), transient over-expression of the *PbrADC1* gene in the epidermal tissue of the ripe ‘Dangshansuli’ fruit led to a higher putrescine content (Fig. 2b-i). On the other hand, an opposite result was observed for the *PbrADC1*-silenced fruit, in which the putrescine level was lower than that in the control (Fig. 2b-ii). A similar result was also observed after transient overexpression of the *PbrADC1* gene in ‘Yali’ fruit (Fig. S6a).

To confirm the role of *PbrADC1* in chilling stress, the *PbrADC1*-overexpressing tomato fruits with stable inheritance were generated (Fig. S7a-i/b-i). As shown in Fig. 2c, a substantial increase in putrescine abundance was observed in the epidermal tissue of transgenic fruits, which was associated with a lower chilling injury index when compared to that of control fruits.

In combination, these results suggested that PbrADC1 prompted fruit putrescine biosynthesis and thus chilling resistance.

### Evolution and characteristics of plant ADCs

In a further study, we assayed if ADC was conserved in plant kingdom. As shown in Fig. S8a and Table S7, a total of 47 ADCs, which originated in plant kingdom approximately 1160 million years ago (MYA), were identified from 26 plants, exhibiting similar physico-chemical characteristics (Fig. S8a and Table S7). Most possessed only one exon (Fig. S8b-ii). Among six motifs identified from plant ADCs, three (Motif 1, 2 & 5) constituted the conserved domain ‘Orn_Arg_deC_N’ (Fig. S8b-iii & c).

**Fig. 3:**
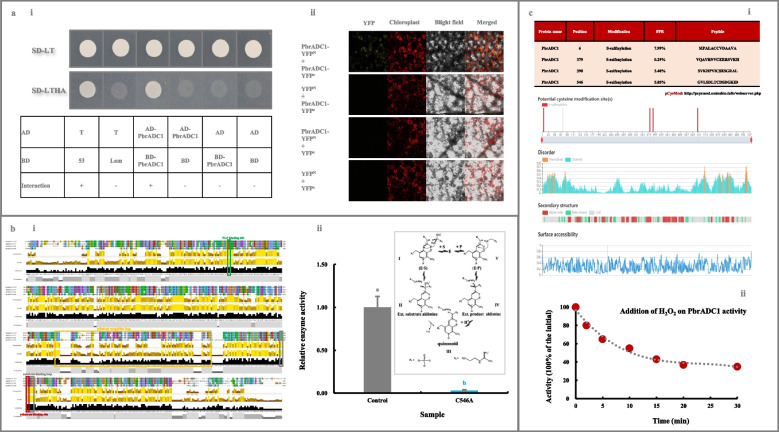
Characteristics of PbrADC1. **a** PbrADC1 self-interaction determination. (a-i) Y2H assay. Transformants containing AD-*T* & BD-*53*, AD-*T* & BD-*Lam*, AD & BD, AD & BD-*PbrADC1*, and AD-*PbrADC1* & BD were used as controls. (a-ii) BiFC assay. Transformants containing YFP^N^ & YFP^C^, YFP^N^ & *PbrADC1*-YFP^C^, and YFP^C^ & *PbrADC1*-YFP^N^ were used as controls. **b** Identification and functional validation of the substrate-binding residue Cys^546^ in PbrADC1. (b-i) Alignment of PbrADC1 with AtADCs and HpADC1 by Jalview Version 2. Information on AtADCs and HpADC1 was summarized by Hanfrey et al. (2001). (b-ii) Functional validation of Cys^546^. Enzyme activities of the His-tagged recombinant PbrADC1 and PbrADC1.^C546A^ proteins were analyzed based on the method of Song et al. (2010). **c** Exogenous H_2_O_2_ treatment on PbrADC1 activity. (c-i) Identification of the H_2_O_2_-modified Cys residues in PbrADC1. The H_2_O_2_-modified Cys residues in PbrADC1 was predicted by pCysMod database (Li et al. 2021b). (c-ii) Impact of exogenous H_2_O_2_ treatment on PbrADC1 activity. The residual activity was expressed as a percentage of the initial (0 min). Data represented the mean value of three biological replicates, and different lowercase letters meant significance between samples (*p* < 0.05)

Notably, a self-interaction was observed for PbrADC1 based on the results of yeast two-hybrid (Y2H) and Bimolecular fluorescence complementation (BiFC) assay (Fig. 3a). After alignment of PbrADC1 with AtADCs & HpADC1 (Hanfrey et al. 2001), a substrate-binding residue, Cys^546^, conserved in all 47 plant ADCs (Fig. S9), was identified in PbrADC1 (Fig. 3b-i). Further investigations revealed that mutation of the Cys^546^ residue almost completely inhibited PbrADC1’s ability to convert arginine into agmatine (Fig. 3b-ii). With the aid of pCysMod database (Li et al. 2021b), the Cys^6^, Cys^379^, Cys^390^, and Cys^546^ residues in PbrADC1 were predicted to be modified by H_2_O_2_ (Fig. 3c-i), which subsequently altered the activity of PbrADC1 as evidenced by exogenous H_2_O_2_ treatment (Fig. 3c-ii).

### Characterization and confirmation of PbrWRKY62 as the upstream regulator of PbrADC1 gene

To explore the upstream regulator of *PbrADC1*, we analyzed the *cis*-acting elements in its promoter sequence, using the PlantCARE database. As displayed in Fig. S10a-b, two W-box elements, three MYB-binding sites, and four G-box elements were identified from the *PbrADC1* promoter. Subsequently, we assayed the expression profiles of the related TFs. As shown in Fig. S10c-i/ii and Table S8 and S9, the expression levels of *PbrWRKY9*/*10*/*12*/*62* and *PbrMYB7*/*8*/*64*/*75*/*107*/*114* displayed extremely strong correlations with *PbrADC1* mRNA abundance during cold storage and development of ‘Dangshansuli’ fruit (absolute correlation coefficient ≥ 0.8). Furthermore, considering the alteration of their transcription levels upon superficial scald development in ‘Yali’ fruit (fold change ≥ 2.0 and FDR < 0.05; Fig. S10c-iii and Table S10), PbrWRKY10, PbrWRKY62, and PbrMYB107 were identified as the upstream regulators of the *PbrADC1* gene (Table S11). Their potential binding sites in the *PbrADC1* promoter were identified with the aid of the PlantRegMap database (Tian et al. 2020) (Table S11). Due to its stronger association with *PbrADC1* during the cold storage of ‘Dangshansuli’ fruit than others (Fig. S10c-i), *PbrWRKY62*, whose expression pattern was validated by RT-qPCR analysis (Fig. S3b), was selected for further study. As shown in Fig. S4a/b-ii, the CDS sequences and the corresponding protein sequences of *PbrWRKY62* in ‘Dangshansuli’ and ‘Yali’ fruits were highly identical.

**Fig. 4:**
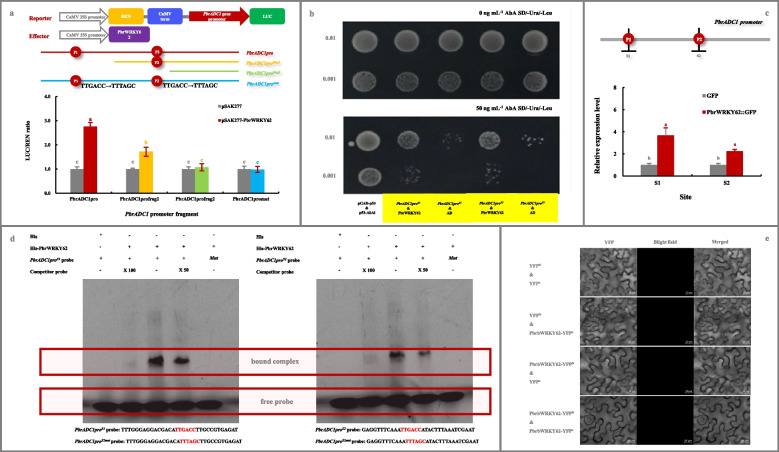
Confirmation of PbrWRKY62 as the upstream regulator of *PbrADC1* gene. **a** Dual-luciferase assay. Transformants containing empty pSAK277 and each report vector were used as controls. **b** Y1H assay. Yeast cell co-transformed with pGAD7-p53 & p53-AbAi was used as a positive control, while yeast cell with pGADT7-AD & *PbrADC1pro*^*S1*^*-*pAbAi and pGADT7-AD & *PbrADC1pro*^S2^-pAbAi, respectively, were used as negative controls. **c** ChIP-qPCR analysis. Pear calli overexpressing the empty pCAMBIA1300-GFP plasmid was used as a negative control. **d** In vitro EMSA assay. FAM luciferase-labelled *PbrADC1* promoter fragments, containing the W-box elements (TTGACC) and its mutant (TTGACC → TTTAGC), were named as *PbrADC1pro*^*S1*^ probe, *PbrADC1pro*^S2^ probe, *PbrADC1pro*^*S1mut*^ probe, and *PbrADC1pro*^*S2mut*^ probe, respectively, and the unlabeled *PbrADC1* promoter fragments containing the W-box elements were used as competitor probes. The presence and absence of His protein, His-PbrWRKY62 protein, labeled probe, or competitor probe were indicated by “ + ” and “ − ”, respectively. Competitor probe concentrations were 50-fold (50 ×) and 100-fold (100 ×) those of the labeled probe. **e** BiFC assay for the self-interaction of PbrWRKY62. Transformants containing YFP^N^ & YFP^C^, YFP^N^ & *PbrWRKY62*-YFP^C^, and YFP^C^ & *PbrWRKY62*-YFP.^N^ were used as controls. Data represented the mean value of three biological replicates, and different lowercase letters meant significance between samples (*p* < 0.05)

Co-transformation of pSAK277-*PbrWRKY62* and reporter (pGreen 0800-*PbrADC1*-LUC vector) resulted in a 2.8-fold increment of the Firefly luciferase/renilla luciferase (LUC/REN) ratio in *N. benthamiana* leaves (Fig. 4a). Such increment was positively correlated with the number of W-box elements (Fig. 4a). However, such increment disappeared after mutation of the two W-box elements (TTGACC → TTTAGC; Fig. 4a). In the yeast one-hybrid (Y1H) assay, yeast cells of positive control (pGADT7-p53 & p53-pAbAi), negative controls (*PbrADC1pro*^*S1*^-pAbAi & pGADT7 and *PbrADC1pro*^*S2*^-pAbAi & pGADT7), and bait-prey (*PbrADC1pro*^*S1*^-pAbAi & pGADT7-*PbrWRKY62* and *PbrADC1pro*^*S2*^-pAbAi & pGADT7-*PbrWRKY62*) grew normally in screening medium (Fig. 4b). When AbA was added, the growth of negative control was inhibited, without affecting on the others (Fig. 4b). Chromatin immunoprecipitation-quantitative PCR (ChIP-qPCR) assay, using an anti-GFP antibody, revealed that the fragments of *PbrADC1* promoter containing W-box elements were enriched in the presence of PbrWRKY62 (Fig. 4c). In vitro electrophoretic mobility shift (EMSA) assay demonstrated that the protein-DNA complexes were observed when the recombinant His-PbrWRKY62 protein was incubated with labeled probes containing the W-box elements. These complexes gradually diminished with the increased amount of the unlabeled competitor probes (Fig. 4d). Furthermore, the complexes disappeared after the mutation of W-box elements (TTGACC → TTTAGC; Fig. 4d). Additionally, no self-interaction was observed for PbrWRKY62 based on the results of BiFC assay (Fig. 4e).

Taken together, these findings indicated that PbrWRKY62 could bind to the two W-box elements in the *PbrADC1* promoter and then activate its transcription as a monomer.

### Functional validation of PbrWRKY62 involved in putrescine biosynthesis and thus chilling resistance

**Fig. 5:**
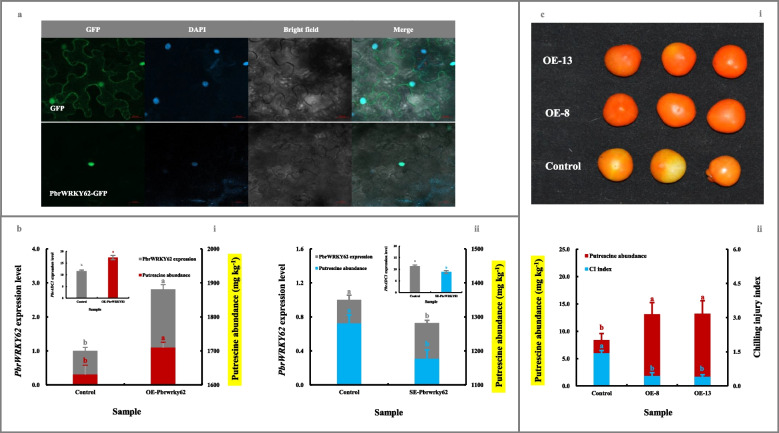
Functional validation of PbrWRKY62 involved in fruit putrescine biosynthesis and thus chilling resistance. **a** Subcellular localization of PbrWRKY62. 4’,6-diamidino-2-phenylindole (DAPI) was used as a nuclear indicator (Kapuscinski 1995). **b** Impact of transient genetic transformation in the ripe ‘Dangshansuli’ fruit on putrescine abundance. (b-i) Transient overexpression of *PbrWRKY62* gene. The ripe ‘Dangshansuli’ fruit transformed with the empty vector was used as a control. (b-ii) Transient silence of *PbrWRKY62* gene. The ripe ‘Dangshansuli’ fruit co-transformed with empty pTRV2 and pTRV1 was used as a control. The expression level of *PbrWRKY62* in control fruit was set as 1.0 for RT-qPCR. **c** Impact of overexpressing *PbrWRKY62* gene in tomato on fruit putrescine biosynthesis and thus chilling resistance. (c-i) Visual quality change. (c-ii) Putrescine content and chilling injury index. Tomato fruits at 35 DAFB, including the wide-type (control) and overexpressing (OE) lines, were harvested and then exposed to 4 ℃ for 10 d followed by 20 ℃ storage for 7 d. Data represented the mean value of three biological replicates, and different lowercase letters meant significance between samples (*p* < 0.05)

As demonstrated in Fig. 5a, PbrWRKY62, without any signal peptide and transmembrane domain (Fig. S5a/b-ii), accumulated in the nucleus of *N. benthamiana* leaves.

To explore the effect of *PbrWRKY62* on putrescine biosynthesis, we analyzed its function in pear fruits. Transient over-expression of *PbrWRKY62* in the epidermal tissue of the ripe ‘Dangshansuli’ fruit substantially promoted *PbrADC1* transcription and thus putrescine accumulation. A similar result was also observed after transient overexpression of the *PbrWRKY62* gene in ‘Yali’ fruit (Fig. S6b). In contrast, a opposite phenomenon was observed in the *PbrWRKY62*-silenced ‘Dangshansuli’ fruit, where *PbrADC1* mRNA abundance and putrescine level were lower than those in the control fruit (Fig. 5b).

To further validate the role of *PbrWRKY62* in the low-temperature stress, the *PbrWRKY62*-overexpressing tomato fruits were generated (Fig. S7a-ii/b-ii). As illustrated in Fig. 5c, transgenic fruits with upregulated putrescine levels showed increased resistance to chilling stress, when compared with that in the control fruits, as evidenced by a lower chilling injury index.

Collectively, these results revealed that PbrWRKY62, as the upstream regulator of *PbrADC1*, could enhance the accumulation of putrescine, and thus increase fruit chilling tolerance.

## Discussion

Superficial scald, a physiological disorder that develops during the cold storage and shelf life of apple and pear fruit, is the result of oxidative damage (Giné-Bordonaba et al. 2020; Qian et al. 2021). Recently, several physico-chemical methods, which demonstrate the potential to suppress ROS accumulation, have been applied to control superficial scald development in fruit, including 1-MCP fumigation, DPA dipping, resveratrol immersion, polyamines treatment, MAP, and CA storage (Calvo et al. 2018; Dias et al. 2020; Feng et al. 2018; Li et al. 2021a; Li et al. 2016; Mditshwa et al. 2017; Poirier et al. 2020; Qian et al. 2021). Consistent with the results of previous reports (Calvo et al. 2018; Li et al. 2021a), putrescine, whose abundance was positively associated with superficial scald severity/incidence/index in ‘Yali’ and ‘Dangshansuli’ fruit (Fig. 1b-c), could mitigate superficial scald development in pear (Fig. 1d).

To explore the molecular mechanism responsible for such phenomenon, we firstly characterized 21 genes in the putrescine-metabolic pathway, with the aid of *P. bretschneideri* Rehd. genome database (Wu et al. 2013) and the transcriptome annotation (Table S2). Subsequently, through a conjoin analysis of metabolites and gene expression profiles during superficial scald development of ‘Dangshansuli’ and ‘Yali’ fruits (Fig. 1a-c and Tables S5 and S6), PbrADC1, located in the chloroplast as homodimer (Figs. 2a & 3a), was experimentally validated as a key player in fruit putrescine biosynthesis and thus chilling resistance (Fig. 2b-c & S6a). The substrate-binding residue Cys^546^ in PbrADC1 was explored to play a critical role in arginine decarboxylation (Fig. 3b). Consistent with these results, AtADC1 and AtADC2, the homologues of PbrADC1 from *Arabidopsis*, could form a hetero-and homodimer, and participate in plant adaption to abiotic environments via regulating putrescine biosynthesis (Maruri-López and Jiménez-Bremont 2017; Urano et al. 2004). Additionally, mutation of the substrate-binding residue (Cys^524^) in AtADC1 almost abolished its activity (Hanfrey et al. 2001). *ADC* from oat (Capell et al. 1998) and *SaADC1* from potato (Kou et al. 2018) displayed similar functions.

Numerous posttranslational H_2_O_2_ modifications have been discovered within proteomes, creating a complex landscape of protein diversity and function (Huang et al. 2019; Waszczak et al. 2014). The cysteinyl thiol in the catalytic residue Cys^20^ of AtDHAR2 could be sulfenylation (SOH) by H_2_O_2_, thereby suppressing its activity (Waszczak et al. 2014). Notably, exogenous H_2_O_2_ treatment inhibited PbrADC1 activity (Fig. 3c-ii). Considering the pCysMod result (Fig. 3c-i), this phenomenon might be due to the S-sulfinylation or S-sulfenylation of several Cys residues in PbrADC1, including Cys^6^, Cys^379^, Cys^390^, and Cys^546^. Cys-SOH could act as a regulatory switch in several oxidative stress signal transduction pathways (Waszczak et al. 2014). Given that H_2_O_2_ accumulated during the cold storage of pear fruit (Gao et al. 2015; Yu et al. 2011), our result suggested that PbrADC1 might be involved in superficial scald development as a regulatory switch.

Until recently, a couple of TFs have been characterized as upstream regulators of *ADC* genes via binding to the corresponding *cis*-acting elements (Gong et al. 2015; Song et al. 2022; Wu et al. 2016). After analyzing the distribution of *cis*-acting elements in the *PbrADC1* promoter and the expression profiles of the corresponding TFs followed by experimental validation, PbrWRKY62 was characterized and experimentally validated as the direct positive transcriptional regulator of the *PbrADC1* gene, enhancing fruit putrescine level and thus chilling tolerance (Figs. 4 and 5, S6b & S10 and Tables S8-S11). Similar roles were reported for CsCBF1 from *Citrus sinensis* (Song et al. 2022) and FcWRKY70 from *F. crassifolia* (Gong et al. 2015), while, PtrNAC72 in *Poncirus trifoliata* exhibited a reverse role (Wu et al. 2016).

**Fig. 6:**
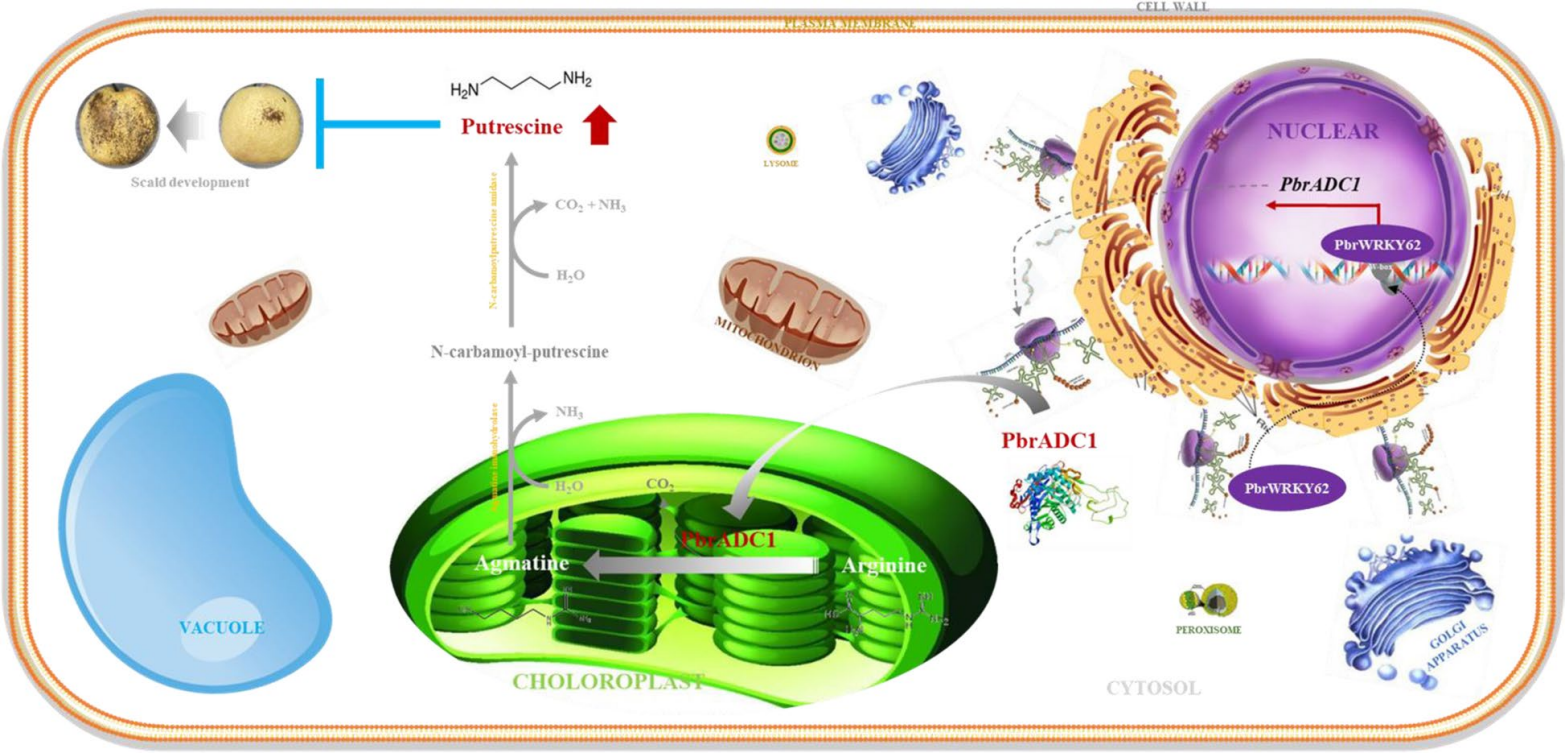
Schematic model of PbrWRKY62 and its downstream target gene *PbrADC1* elevating fruit putrescine level and thus chilling tolerance. PbrWRKY62, located in the nucleus, could interact with two W-box elements in *PbrADC1* promoter as monomer and then activate its expression. After translation in the ribosome, PbrADC1 was then transported into the chloroplast, where it converted arginine to agmatine, elevating putrescine level and thus chilling resistance of fruit

Overall, our results suggest that the PbrWRKY62-PbrADC1 module enhances fruit chilling tolerance via regulating putrescine biosynthesis. Therefore, they could serve as the candidates for breeding superficial scald-resistant pear fruit. Figure 6 illustrates the schematic model: PbrWRKY62, located in the nucleus, interacts with two W-box elements in the *PbrADC1* promoter as a monomer, activating the expression of *PbrADC1*. After translation in the ribosome, PbrADC1 is transported into the chloroplast, where it converts arginine to agmatine, increasing putrescine level and thereby enhancing the chilling resistance of fruit.

## Conclusion

In this study, we experimentally validated that PbrADC1, located in the chloroplast as a homodimer, played a key role in superficial scald development via regulating putrescine biosynthesis. The substrate-binding residue Cys^546^ in PbrADC1, whose activity could be suppressed by H_2_O_2_, was found to be crucial for arginine decarboxylation. Additionally, the nuclear PbrWRKY62 could act as the direct upstream regulator of *PbrADC1*, enhancing putrescine accumulation and thus improving chilling tolerance in fruit. Taken together, our results suggest that PbrWRKY62-PbrADC1 module is involved in superficial scald development in pear fruit via regulating putrescine biosynthesis.

## Materials and methods

### Plant material

#### Experiment I

Uniform and defect-free *P. bretschneideri* Rehd. cv. ‘Yali’ fruits, harvested from homogeneous trees in an orchard in Xinji City, Hebei Province on September 14, 2016, were stored at -0.5 ℃ for 180 days before a 7-day shelf life at 20 ℃. Fruits with and without superficial scald were sampled for further analysis.

#### Experiment II

*P. bretschneideri* Rehd. cv. ‘Dangshansuli’ fruits, harvested from homogeneous trees in an orchard in Pucheng County, Shaanxi Province on September 25, 2016, were stored at 0.5 ℃ for 180 days. Fruits were sampled every 60 days, followed by a 7-d shelf life at 25 ℃.

#### Experiment III

‘Yali’ fruits, harvested from homogeneous trees in an orchard in Xinji City, Hebei Province on September 14, 2017, were stored at -0.5 ℃ for 180 days before a 7-d shelf life at 20 ℃. Fruit with different superficial scald severities (Hui et al. 2016) were sampled for further analysis.

#### Experiment IV

‘Yali’ fruits, harvested from homogeneous trees in an orchard in Gaoyou City, Jiangsu Province on September 10, 2018, were randomly divided into three groups for different treatments before storage at -0.5 ℃: 0.0 (H_2_O, control), 2.0-, and 5.0-mM putrescine immersion for 15 min. Fruits were sampled on day 210, followed by a 7-d shelf life at 20 ℃.

In this study, ‘Dangshansuli’ and ‘Yali’ fruits were preserved in cold storage and on shelves at different temperatures to simulate their environment during the marketing period in Shannxi and Hebei provinces. Considering the result of Hui et al. (2016), superficial scald symptom, which might not be visible during chilling storage, appeared or aggravated during shelf life at ambient temperature. Therefore, in this study, we stored the fruit at a low temperature followed by a 7-d shelf life to facilitate superficial scald development. There were three biological replicates for each treatment. For sampling, the epidermal tissue from five fruit per replicate was quickly removed with a brass cork borer, mixed, and then stored at -80 ℃ for further assays.

### Superficial scald incidence and index analysis

Based on the superficial scald area in the epidermal tissue, superficial scald severities were assigned as follows: Severity 0: no superficial scald; Severity 1: superficial scald area < 25%; Severity 2: 25% ≤ superficial scald area < 50%; Severity 3: superficial scald area ≥ 50% (Hui et al. 2016). Superficial scald incidence and severity were calculated using the following formulas (He et al. 2022; Qian et al. 2021):

Superficial scald incidence = Number of scalded fruit / Total number of fruit × 100%

Superficial scald index = ∑(Number of fruit × severity) / (Total number of fruit × 3).

### Metabolite measurement

Polyamines, including putrescine, spermine, and spermidine, were extracted from epidermal tissue and analyzed using an RIGOL L-3000 high-performance liquid chromatography (HPLC) system (Rigol, Beijing, China), equipped with a Rigol C18 reversed-phase column and an RF-20A detector (Marcé et al. 1995). In brief, 0.1 g tissue was homogenized with 1.0 mL of 5% HClO_4_, ultrasonicated for 30 min, and then centrifuged at 10,000 × *g* for 10 min. Subsequently, 60 μL of 2 M NaOH, 60 μL of saturated NaHCO_3_, and 200 μL of 10 g L^−1^ dansyl chloride were added, respectively, to the supernatant, followed by incubation in 50 ℃ hot water for 40 min. After cooling to 4 ℃, 100 μL of ammonia was added to the sample. It was then placed at 25 ℃ for 30 min, settled to a volume of 1.0 mL with methanol, and filtered through a 0.22 μm membrane before polyamine determination by the HPLC system.

Amino acids, including arginine, ornithine, and citrulline, were extracted and analyzed following the protocol of Sánchez-Machado et al. (2003), with some modifications. In brief, 0.2 g tissue was homogenized with 1.0 mL of ultrapure water. After incubation overnight and then filtration through a 0.22 μm membrane, amino acids were derivatized and assayed using an HPLC (Rigol L3000-system, Rigol, Beijing, China) equipped with a Sepax Bio-C18 reversed-phase column and a UV detector.

Fumarate in the epidermal tissue was extracted with ultrapure water. After centrifugation, the supernatant was filtered through a 0.22 μm membrane before analysis by an HPLC (Rigol L3000-system, Rigol, Beijing, China) equipped with a Sepax Bio-C18 reversed-phase column and a UV detector. NO was extracted and measured following the protocol of the NO assay kit (NO-1-G, Suzhou Comin Biotechnology Co., Ltd., China).

### Transcriptome and RT-qPCR analysis

Transcriptome assay was conducted following the protocol described by Li et al. (2019b). Total RNA was extracted from the epidermal tissue of *P. bretschneideri* Rehd. fruit using the RNAprep pure Plant Kit (Tiangen, Beijing, China). Subsequently, an aliquot of total RNA was treated with DNase (Takara, Dalian, China) to remove DNA before checking RNA integrity, concentration and purity via NanoDrop 2000 (Thermo, CA, USA) and the Agilent Bioanalyzer 2100 system (Agilent, CA, USA). Next, 5.0 μg RNA was used to construct a complementary DNA (cDNA) library, which was subsequently sequenced using the BGISEQ-500 platform (BGI, Shenzhen, China). After removing adapter sequences and low-quality reads, the clean reads were aligned to the *P. bretschneideri* Rehd. Genome database (Wu et al. 2013), using the HISAT and Bowtie2 tools (Li et al. 2019a). Gene expression was quantified as Fragments Per Kilobase Million (FPKM), and differentially expressed genes (DEGs) were identified with the NOISeq software based on the following criteria: fold change ≥ 2.0 and FDR < 0.05 (Li et al. 2019a). For the transcriptome assay of gene expression profiles in the epidermal tissue of ‘Dangshansuli’ fruit, there were three biological replicates, which were sampled after 0-/60-/120-/180-d low-temperature storage, followed by a 7-d shelf life. For the transcriptome assay of gene expression profiles in the epidermis of (un)scalded ‘Yali’ fruit, there were two biological replicates. For the transcriptome assay of gene expression profiles in the cortex of the developing ‘Dangshansuli’ fruit, there was one biological replicate at six stages: fruit-setting stage (15 days after full blooming (15 DAFB)), physiological fruit dropping stage (34 DAFB), a month after fruit enlargement stage (81 DAFB), pre-mature stage (110 DAFB), mature stage (145 DAFB), and fruit senescence stage (160 DAFB).

For the RT-qPCR assay, gene-specific primers were designed using Primer 5.0 software (Table S1). Total RNA was isolated using Trizol Reagents (Invitrogen, USA) and treated with RNase-free DNase (Qiagen, USA). After analyzing RNA integrity, concentration, and purity, first-strand cDNA synthesis was carried out using TransScript® One-Step gDNA Removal and cDNA Synthesis SuperMix (TRANSGEN, China). Finally, RT-qPCR assays were performed using the SYBR® PrimeScript™ RT-PCR Kit (Perfect Real Time; Takara) in a 10-μL reaction volume, which included 5.0 μL of LightCycler 480 SYBR GREEN I Master, 1.0 μL of 10 μmol L^−1^ forward and reverse primers, 1.0 μL of 100 ng μL^−1^ cDNA, and 3.0 μL of RNase-free water (Wang et al. 2018). The *Pyrus tubulin* (*PbrTub*) gene served as the internal reference gene, and the relative gene expression level was calculated using the 2^−ΔΔCT^ method (Wang et al. 2018).

### Alignment of PbrADC1 and PbrWRKY62 coding sequences (CDSs) and protein sequences from different pear cultivars

The CDSs of *PbrADC1* and *PbrWRKY62* genes were amplified from ‘Dangshansuli’ and ‘Yali’ fruits using the method described by Ma et al. (2020). The corresponding protein sequences were deduced from their CDSs, using the Translate database (https://web.expasy.org/translate/). Sequence alignment was performed by Jalview Version 2 (Waterhouse et al. 2009).

### Subcellular localization assay

For the subcellular localization of PbrADC1 and PbrWRKY62, their CDSs encoding mature proteins were amplified from ‘Dangshansuli’ fruit (Table S1), and then inserted into the pBI221 vector which was fused with a GFP tag. The recombinant plasmid was subsequently introduced into *N. benthamiana* leaves following the protocol of Lin et al. (2022).

The fluorescence signal was detected using a confocal microscope (LSM510 Meta, Zeiss, Germany). GFP fluorescence was observed with an excitation wavelength of 488 nm and an emission wavelength of 495–550 nm, while chloroplast autofluorescence was observed under an excitation wavelength of 488 nm and emission wavelength of 664–696 nm (Chabregas et al. 2003). 4’,6-diamidino-2-phenylindole (DAPI) was used as a nuclear marker (Kapuscinski 1995).

### Determination of PbrADC1 and PbrADC1^C584A^ (substitution of Cys^584^ residue in PbrADC1 with Ala^584^) activity in vitro

The CDS of *PbrADC1* was amplified from ‘Dangshansuli’ fruit (Table S1), and then inserted into the pCold-TF plasmid. Subsequently, the recombinant plasmid was introduced into *E. coli* BL21 (DE3) to express the His-tagged fusion protein (Ma et al. 2020). After purification through a Ni–NTA His Bind resin column (Sangon, Shanghai, China), the recombinant His-PbrADC1 protein was collected for further analysis (Ma et al. 2020). To introduce the C584A point mutation into the *PbrADC1* CDS, the pCold-TF::PbrADC1 plasmid was used as a template, and the mutation was achieved by overlapping PCR with the mutagenic primers (Table S1) (Do et al. 2016). After confirmation of the mutation by DNA sequencing, the constructed plasmids were then transformed into *E. coli* BL21 (DE3) for protein expression and purified based on the protocol used for PbrADC1 (Ma et al. 2020). In vitro PbrADC1 and PbrADC1^C584A^ activity were determined spectrophotometrically by monitoring agmatine formation (Song et al. 2010).

The impact of exogenous H_2_O_2_ supplementation on PbrADC1 activity was assessed using methods from previous reports (Navarre et al. 2000; Verniquet et al. 1991). The His-tagged recombinant PbrADC1 protein was incubated with 100 μM H_2_O_2_ for 0, 2, 5, 10, 15, 20, and 30 min. After removing the excess H_2_O_2_ with the Micro Bio-Spin P-6 gel column (BioRad), the residual activity was assayed by monitoring agmatine formation (Song et al. 2010); and the result was expressed as a percentage of the initial (0 min).

### Gene function validation in vivo

#### Transient overexpression of genes in pear

*PbrADC1* and *PbrWRKY62* ORFs, after amplification from ‘Dangshansuli’ fruit, were inserted into the pCAMBIA1300 vector with a GFP tag (Table S1). These constructs were then transformed into *A. tumefaciens* strain GV3101, respectively, and incubated at 28 °C until OD_660_ reached 1.0. After resuspending the bacterial strain in the infiltration buffer (10 mmol L^−1^ MgCl_2_, 10 mmol L^−1^ MES, and 150 μmol L^−1^ acetosyringone), 5 μL of the solution was slowly and vertically injected into each point in the epidermal tissue of the ripe ‘Dangshansuli’ and ‘Yali’ fruits using a needleless syringe (Jiang et al. 2019; Li et al. 2012; Ma et al. 2020). After 3-d storage in the dark at 25 °C, the epidermis from each injection site was sampled. Fruits infiltrated with an empty vector were used as controls. There were three biological replicates per treatment, with eight fruit per biological replicate.

#### Transient silencing of genes in pear

Approximately 200-bp fragments of *PbrADC1* and *PbrWRKY62* ORFs were amplified from the ‘Dangshansuli’ fruit and then inserted into the pTRV2 vector (Table S1). The recombinant plasmid and pTRV1 were transformed into *A. tumefaciens* strain GV3101, respectively. Afterward, the bacterial resuspensions containing recombinant pTRV2 and pTRV1 were mixed in a 1:1 ratio before the slow and vertical injection into the epidermal tissue of the ripe ‘Dangshansuli’ fruit, using a needleless syringe (Jiang et al. 2019; Li et al. 2012; Zhang et al. 2019). After 3-d of storage in the dark at 25 °C, the epidermis from each injection site was sampled. Fruits co-injected with the empty pTRV2 vector and pTRV1 were used as a control. There were three biological replicates per treatment, with eight fruit per biological replicate.

#### Transformation of tomato

After the construction of *PbrADC1*/*PbrWRKY62-*overexpressing vectors as mentioned above, the transformation of *S. lycopersicum* cv. MicroTom was carried out based on the method of Cheng et al. (2018), using leaf disk or epicotyl as an explant. Positive transgenic lines were screened by kanamycin (100 mg L^−1^) selection and then confirmed at the DNA level by PCR and RNA level by RT-qPCR. All plants were grown in a greenhouse (25 ℃ light for 18 h /18 ℃ dark for 6 h, 60% relative humidity).

One typical chilling symptom of tomato fruit is uneven ripening, which manifests as abnormal color development (Liu et al. 2020). To assess gene function in chilling stress, tomato fruits from the wild-type (control) and homozygous lines (T2 generation) were harvested at 35 DAFB and then stored at 4 ℃ for 10 days followed by a 7-d shelf life at 20 ℃. The chilling injury index of tomato fruit was calculated based on the level of uneven ripening (a five-point scale based on the ripening stage for each criterion: 0 = red, 1 = orange, 2 = yellow, 3 = breaker, 4 = green) (Liu et al. 2020).

### DNA and protein interaction

#### Dual-luciferase reporter assay

*PbrWRKY62* ORF was introduced into the pSAK277 vector, while different fragments of the *PbrADC1* promoter, containing various numbers of the W-box elements (*PbrADC1pro* and *PbrADC1pro*^*frag1/frag2*^) or the mutated elements (TTGACC → TTTAGC, *PbrADC1pro*^*mut*^), were inserted into the pGreen 0800-LUC vector to create various reporters (Table S1). Subsequently, a mixture of *A. tumefaciens* containing pSAK277-*PbrWRKY62* and each reporter was infiltrated into *N. benthamiana* leaves (Liu et al. 2022). Transformants containing the empty pSAK277 vector and each reporter were used as controls. LUC and REN activities were determined using a dual-luciferase reporter assay system (Promega, Madison, WI, USA).

#### Y1H assay

*PbrWRKY62* CDS was amplified and inserted into the prey vector pGADT7 (AD), while approximately 200-bp fragments of the *PbrADC1* promoter, each containing a W-box element, were inserted into the bait vector pAbAi (Table S1). The Y1H assay was conducted using the Matchmaker Gold Yeast One-Hybrid Library Screening System (Weidi, Shanghai, China) (Liu et al. 2019). SD/-Ura medium, supplemented with various AbA concentrations, was used to check the self-activation of *PbrADC1pro*^*S1/S2*^-pAbAi and then select the appropriate AbA concentration.

#### ChIP-qPCR

ChIP-qPCR assay following the method of Li et al. (2022), with some modifications. The pCAMBIA1300-*PbrWRKY62-GFP* plasmid, constructed as mentioned above (transient overexpression of genes in pear), was transformed into calli induced from fruitlets of *P. communis* cv. ‘Clapp’s Favorite’ pear (Ni et al. 2021). Calli overexpressing the empty vector were used as a negative control. Subsequently, *PbrWRKY62*-overexpressing calli and the negative control were used for cross-linking DNA and protein with 1% (v/v) formaldehyde. After homogenization and cell lysis, chromatin was obtained and then sonicated to obtain soluble sheared chromatin with an average DNA length of 200–500 bp. One part of the soluble sheared chromatin was used for input DNA, and the other for immunoprecipitation using an anti-GFP antibody (Li et al. 2018). The enrichment of *PbrADC1* promoter fragments was evaluated by qPCR (Table S1).

#### EMSA assay

The His-tagged PbrWRKY62 protein was obtained based on the method as described by Ma et al. (2020). 33-bp FAM luciferase-labeled DNA probes, containing either the wild-type or mutated W-box elements, as well as the unlabeled competitor probes, were synthesized with the assistance of Genewiz, Inc. (Suzhou, China). EMSA binding reactions were performed conducted following the manufacturer's instructions (LightShift EMSA Kit; Thermo Scientific) (Zhu et al. 2021).

### Protein self-interaction

#### Y2H assay

*PbrADC1* CDS was cloned into pGADT7 (AD) and pGBKT7 (BD) vectors (Table S1). Afterwards, these constructs were co-transformed into the *S. cerevisiae* strain AH109, and then dripped on the synthetic dropout nutrient media SD/-Leu/-Trp (SD-LT) and SD/-Leu/-Trp/-His/-Ade (SD-LTHA) (Ma et al. 2020). Transformants containing AD-*T* & BD-*53*, AD-*T* & BD-*Lam*, AD & BD, AD & BD-*PbrADC1*, and AD-*PbrADC1* & BD were used as controls.

#### BiFC analysis

The CDSs encoding the mature proteins of *PbrADC1* and *PbrWRKY62* were amplified from ‘Dangshansuli’ fruit (Table S1), and inserted into 35S-pSPYNE-YFP^N^ (YFP^N^) and 35S-pSPYCE-YFP^C^ (YFP^C^). These recombinant constructs were introduced into the *A. tumefaciens* strain GV3101, and then infiltrated into *N. benthamiana* leaves. YFP fluorescence signal was detected using a confocal laser scanning microscope (LSM510 Meta, Zeiss, Germany) (Ma et al. 2020). Transformants containing YFP^N^ & YFP^C^, YFP^N^ & *PbrADC1/PbrWRKY62*-YFP^C^, YFP^C^ & *PbrADC1/PbrWRKY62*-YFP^N^ were used as controls.

### In silico analysis

The protein sequences of the putrescine-metabolism-related genes in *Arabidopsis* genome were obtained from The *Arabidopsis* Information Resource (TAIR) database (http://www.arabidopsis.org/) (there is no *ODC* gene in its genome) (Table S2). These sequences were used as queries to perform BLASTP searches against the *P. bretschneideri* Rehd. genome database (http://peargenome.njau.edu.cn/) (Wu et al. 2013). Subsequently, all candidate genes were then submitted to Pfam (http://pfam-legacy.xfam.org/) and SMART (http://smart.embl-heidelberg.de/) databases for verification of the presence of conserved domains (Zhang et al. 2021). Similar methods were employed to identify *ADC* genes from other plants using the Phytozome database (http://www.phytozome.net) (Goodstein et al. 2012).

Physiol-biochemical parameters were calculated using the ProtParam tool (https://web.expasy.org/protparam/) (Zhang et al. 2021). The timescale tree was generated via TIMETREE (http://www.timetree.org/) (Kumar et al. 2017); and phylogenetic trees were constructed using the MEGA7.0 software with the maximum likelihood (ML) method and bootstrap analysis with 1000 replicates (Ma et al. 2020). Gene structures were visualized via the Gene Structure Display Server (http://gsds.cbi.pku.edu.cn/) (Hu et al. 2015), while *cis*-acting elements and motifs were identified using the PlantCARE (http://bioinformatics.psb.ugent.be/webtools/ plantcare/html/) (Lescot et al. 2002) and MEME Suite (https://meme-suite.org/meme/index.html) (Bailey et al. 2015) databases, respectively. Signal peptides were predicted using the SignalP 5.0 database (https://services.healthtech.dtu.dk/services/SignalP-6.0/) (Teufel et al. 2022), and transmembrane helices were characterized using the DeepTMHMM database (https://dtu.biolib.com/DeepTMHMM) (Hallgren et al. 2022). The binding sites of transcription factors (TFs) in the *PbrADC1* promoter were identified using the PlantRegMap database (http://plantregmap.gao-lab.org/) (Tian et al. 2020). Cysteine (Cys) modification sites and types were predicted using the pCysMod database (http://pcysmod.omicsbio.info/webserver.php) (Li et al. 2021b).

Gene chromosomal locations were determined based on genome annotation and visualized using Circos (Krzywinski et al. 2009). The syntenic relationships among genes were analyzed using a method similar to that used for PGDD, and duplicated genes were categorized into the following types: WGD/segmental, tandem, singleton, proximal, and dispersed (Zhang et al. 2019). The Ka and Ks substitution rates of syntenic gene pairs were annotated using MCScanX downstream analysis tools; and the KaKs Calculator 2.0 was used to determine Ka and Ks with the Nei-Gojobori (NG) method (Zhang et al. 2019).

### Statistical analysis

The data presented in this study represent the mean values of three biological replicates, except for the transcriptome assay for both the (un)scalded ‘Yali’ fruit (two replicates) and the developing ‘Dangshansuli’ fruit (one replicate). Data analysis was performed using SAS version 9.3 (SAS Institute, Cary, NC), specifically utilizing analysis of variance (PROC ANOVA) with multi-comparison correction. Mean separation was determined through pairwise comparison using Duncan’s multiple range test at the 0.05 significance level. Pearson correlation coefficients between attributes were calculated using R software, with extremely strong correlations falling within the range of 0.8–1.0 and strong correlations within the range of 0.6–0.8 (Long et al. 2014).

### Supplementary Information

**Additional file 1:** **Fig. S1. **Putrescine metabolic pathway in plants (Kou et al. 2018; Winter et al. 2015). Putrescine in plant could be synthesized either from the decarboxylation of ornithine by ornithine decarboxylase (ODC) or from arginine decarboxylase (ADC) pathway, which is consisted of three enzymes: ADC, agmatine iminohydrolase (AIH), and *N*-carbamoylputrescine amidase (NLP) (Kou et al. 2018). Upon formation, putrescine could be converted into spermidine and spermine by spermidine synthase (SPDS) and spermine synthase (SPMS) (Winter et al. 2015). On the other hand, ornithine and arginine could be interconverted with the aid of ornithine transcarbamylase (OTC), arginosuccinate synthase (ASS), arginosuccinate lyase (ASL), nitric oxide synthase-like (NOS), and arginase (ARG) (Winter et al. 2015).

**Additional file 2: Fig. S2. **Information on 21 putrescine-metabolism-related genes in the *P. bretschneideri* Rehd. genome. (a) Gene localizations. Chromosome numbers were indicated on the inner side, and different colors represented different chromosomes. Additionally, genes that underwent WGD/segmental duplications were connected with red lines. (b) Syntenic relationship of the WGD/segmental duplicated gene pairs. Chromosome/scaffold segments were indicated by grey horizontal lines, and the broad lines with green/blue color represented genes and its transcriptional orientations. Target genes were marked in red color. WGD/segmental duplication gene pairs were connected with bands. (c) Gene structures and the distribution of *cis*-acting elements. (c-i) Phylogenetic tree. (c-ii) Gene structures. Yellow boxes represent the exons, blue boxes represent the UTRs, while black lines represent the introns. (c-iii) The distribution of *cis*-acting elements. Boxes with distinct colors represent the different *cis*-acting elements. 21 putrescine-metabolism-related genes in pear genome were summarized in Table S2.

**Additional file 3: Fig. S3. **RT-qPCR validation of the expression patterns of *PbrADC1* (a) and* PbrWRKY62* (b). ‘Dangshansuli’ fruit were sampled every 60-d storage at 0.5 ℃ followed by a 7-d shelf life at 25 ℃. Data represented the mean value of three biological replicates. The expression abundances of *PbrADC1* and* PbrWRKY62 *genes in the 0-d fruit were set as 1.0.

**Additional file 4: Fig. S4. **Alignment of CDS sequences and the corresponding protein sequences of *PbrADC1 *and *PbrWRKY62* in ‘Dangshansuli’ and ‘Yali’ fruits. (a) CDS sequences of *PbrADC1 *(a-i) and *PbrWRKY62* (a-ii) genes. (b) Protein sequences of PbrADC1 (b-i) and PbrWRKY62 (b-ii).

**Additional file 5: Fig. S5. **Transmembrane helix and signal peptide assay of PbrADC1 and PbrWRKY62. (a) Transmembrane helix in PbrADC1 (a-i) and PbrWRKY62 (a-ii). (b) Signal peptide in PbrADC1 (b-i) and PbrWRKY62 (b-ii).

**Additional file 6: Fig. S6. **Impact of transient genetic transformation of the ripe ‘Yali’ fruit on putrescine abundance. (a) Transient overexpression of *PbrADC1* gene. (b) Transient overexpression of *PbrWRKY62* gene. The ripe ‘Yali’ fruit transformed with the empty vector was used as a control. The expression abundance of *PbrADC1* or *PbrWRKY62* in the control fruit was set as 1.0 for RT-qPCR assay. Data represented the mean value of three biological replicates, and different lowercase letters meant significance between samples (*p* < 0.05).

**Additional file 7: Fig. S7. **Identification of positive transgenic tomato lines at the DNA and RNA levels. (a) PCR assay of the *PbrADC1*-overexpressing lines (a-i) and *PbrWRKY62*-overexpressing lines (a-ii) at DNA level. (b) Expression profiles of *PbrADC1* (b-i) and *PbrWRKY62* (b-ii) genes in tomato fruits. Tomato fruit at 35 DAFB were harvested and then exposed to 4 ℃ for 10 d followed by 20 ℃ storage for 7 d. Data represented the mean value of three biological replicates, and different lowercase letters meant significance between samples (*p* < 0.05).

**Additional file 8: Fig. S8. **Evolution and characteristics of 47 plant ADCs. (a) Timescale tree of 26 plants drawn by TIMETREE. (b) Gene structures and conserved motifs of 47 plant ADCs. (b-i) Phylogenetic tree. (b-ii) Gene structures. Yellow boxes represent the exons, blue boxes indicate the UTRs, while black lines represent the introns. (b-iii) Conserved motifs. Boxes with distinct colors represent the different motifs. (c) Detailed information on the conserved motifs in plant ADCs. Six conserved motifs were characterized from 47 plant ADCs. Motif 1, 2 & 5 composed the conserved domain ‘Orn_Arg_deC_N’, while Motif 3 & 4 composed the domain ‘d7odca2’. 47 plant ADCs, which were identified from 26 plants, were summarized in Table S7.

**Additional file 9: Fig. S9. **Alignment of plant ADCs by Jalview Version 2. 47 plant ADCs, which were identified from 26 plants, were summarized in Table S7. The substrate-binding residues were highlighted in the red box. Just part of the result was demonstrated.

**Additional file 10: Fig. S10.** Characterization of transcript factors (TFs) possibly regulating the expression of *PbrADC1*. (a) Schematic model of the distribution of *cis*-acting elements in the *PbrADC1 *promoter. Red, green, and blue ellipses represent the W-box elements, G-box elements, and MYB-binding sites, respectively. (b) Detailed information of *cis*-acting elements in *PbrADC1 *promoter. The conserved nucleotide acids were highlighted in red. (c) Expression profiles of TFs and their correlations with *PbrADC1* mRNA abundance. (c-i) During the cold storage of ‘Dangshansuli’ fruit. ‘Dangshansuli’ fruit were sampled every 60-d storage at 0.5 ℃ followed by a 7-d shelf life at 25 ℃. Data, adapted from transcriptome assay, represented the mean values of three biological replicates. (c-ii) During ‘Dangshansuli’ fruit development. ‘Dangshansuli’ fruit were sampled at six developmental stages, including fruit-setting stage (15 DAFB), physiological fruit dropping stage (34 DAFB), a month after fruit enlargement stage (81 DAFB), pre-mature stage (110 DAFB), mature stage (145 DAFB), and fruit senescence stage (160 DAFB). Data, adapted from transcriptome assay of the previous study (Zhang et al. 2021), represented the mean value of one biological replicate. (c-iii) Upon superficial scald development in ‘Yali’ fruit. ‘Yali’ fruits, with and without superficial scald, were sampled after -0.5 ℃ for 180 d followed by a 7-d shelf life at 20 ℃.Data, adapted from transcriptome assay, represented the mean values of two biological replicates.*PbrWRKYs*, *PbrbZIPs*, and *PbrMYBs *genes were characterized from the *P. bretschneideri* Rehd. genome (Cao et al. 2016; Huang et al. 2015; Ma et al. 2021). Color scale represents normalized log2-transformed (FPKM + 1), where red indicates a high level, blue represents a low level, and white indicates a medium level. Absolute correlation coefficients between *PbrADC1* and TFs ≥ 0.8 were visualized in the heatmap, where red lines demonstrated extremely strong positive correlations, while green demonstrated extremely strong negative associations*.*

**Additional file 11: Table S1. **Primers used in this study.

**Additional file 12: Table S2. **Physico-chemical characteristics of 21 putrescine-metabolism-related genes in the *Arabidopsis* and *P. bretschneideri* Rehd. genome. The putrescine-metabolism-related genes were characterized from pear by the BLASTP of P. *bretschneideri* Rehd. genome (Wu et al. 2016) and transcriptome annotation.

**Additional file 13: Table S3. **Duplication types of the putrescine-metabolism-related genes in *P. bretschneideri* Rehd. genome.

**Additional file 14: Table S4. **Ka/Ks ratios of paralogous genes in the *P. bretschneideri* Rehd. genome.

**Additional file 15: Table S5. **Expression profiles (FPKMs) of the putrescine-metabolism-related genes upon superficial scald development in ‘Yali’ fruit. ‘Yali’ fruits, with and without superficial scald, were sampled after -0.5 ℃ for 180 d followed by a 7-d shelf life at 20 ℃. Data, adapted from transcriptome assay, represented the mean values of two biological replicates.

**Additional file 16: Table S6. **Expression profiles (FPKMs) of the putrescine-metabolism-related genes during the cold storage of ‘Dangshansuli’ fruit. ‘Dangshansuli’ fruit were sampled every 60-d storage at 0.5 ℃ followed by a 7-d shelf life at 25 ℃. Data, adapted from transcriptome assay, represented the mean values of three biological replicates.

**Additional file 17: Table S7. **Physico-chemical characteristics of 47 plant ADCs. 47 *ADCs *genes were characterized from 26 plants by the BLASTP of their genome (Goodstein et al. 2012).

**Additional file 18: Table S8. **Expression profiles (FPKMs) of *PbrWRKYs*, *PbrbZIPs*, and *PbrMYBs* during cold storage of ‘Dangshansuli’ fruit. ‘Dangshansuli’ fruit were sampled every 60-d storage at 0.5 ℃ followed by a 7-d shelf life at 25 ℃. *PbrWRKYs*, *PbrbZIPs*, and *PbrMYBs* genes were characterized from the P. bretschneideri Rehd. genome (Cao et al. 2016; Huang et al. 2015; Ma et al. 2021). Data, adapted from transcriptome assay, represented the mean values of three biological replicates.

**Additional file 19: Table S9. **Expression profiles (FPKMs) of *PbrADC1*, *PbrWRKYs*, *PbrbZIPs*, and *PbrMYBs* during ‘Dangshansuli’ fruit development. ‘Dangshansuli’ fruit were sampled at six developmental stages, including fruit-setting stage (15 DAFB), physiological fruit dropping stage (34 DAFB), a month after fruit enlargement stage (81 DAFB), pre-mature stage (110 DAFB), mature stage (145 DAFB), and fruit senescence stage (160 DAFB). Data, which was adapted from transcriptome assay of the previous study (Zhang et al. 2021), represented the mean value of one biological replicate.

**Additional file 20: Table S10. **Expression profiles (FPKMs) of *PbrWRKYs*, PbrbZIPs, and PbrMYBs upon superficial scald development in ‘Yali’ fruit. ‘Yali’ fruits, with and without superficial scald, were sampled after -0.5 ℃ for 180 d followed by a 7-d shelf life at 20 ℃. *PbrWRKYs*, *PbrbZIPs*, and *PbrMYBs* genes were characterized from *P. bretschneideri* Rehd. genome (Cao et al. 2016; Huang et al. 2015; Ma et al. 2021). Data, adapted from transcriptome assay, represented the mean values of two biological replicates.

**Additional file 21: Table S11. **Possible binding sites of *PbrWRKY10*, 62 and *PbrMYB107* in the *PbrADC1* promoter. Information on the W-box elements, G-box elements, and MYB-binding sites in *PbrADC1* promoter was summarized in Fig. S10a-b. The possible binding sites of *PbrWRKY10*, 62 and *PbrMYB107* in *PbrADC1* promoter were identified, with the aid of PlantRegMap database (Tian et al. 2020).

## Data Availability

Transcriptome assay was conducted by BGI Gene Tech Co., Ltd. (Shenzhen, China) to analyze gene expression profiles (FPKMs) during cold storage of ‘Dangshansuli’ fruit as well as upon superficial scald development in ‘Yali’ fruit; on the other hand, transcriptome assay was conducted by Biomarker Technologies Co, LTD (Beijing, China) to analyze gene expression profiles (FPKMs) during ‘Dangshansuli’ fruit development. The raw sequence data reported in this paper have been deposited in the Genome Sequence Archive (Genomics, Proteomics & Bioinformatics 2021) in National Genomics Data Center (Nuclear Acids Res 2022), China National Center for Bioinformation/Beijing Institute of Genomics, Chinese Academy of Sciences (GSA: CRA011352, CRA011265, and CRA011138). All data generated or analyzed during this study are included in this published article and its supplementary information files (Tables S5-S6 & S8-S10). Moreover, all other data are available from the corresponding author upon reasonable request.

## Abbreviations

ADC: Arginine decarboxylase
AIH: Agmatine iminohydrolase
ARG: Arginase
ASL: Arginosuccinate lyase
ASS: Arginosuccinate synthase
BiFC: Bimolecular fluorescence complementation
CA: Controlled atmosphere
CAT: Catalase
CDSs: Coding sequences
ChIP-qPCR: Chromatin immunoprecipitation -quantitative qPCR
CTols: Conjugated trienols
Cys: Cystein
DAFB: Days after full blooming
DAPI: 4’,6-Diamidino-2-phenylindole
DEGs: Differentially expressed genes
DPA: Diphenylamine
EMSA: Electrophoretic mobility shift
FPKM: Fragments Per Kilobase Million
H_2_O_2_: Hydrogen peroxide
HPLC: High-performance liquid chromatography
LUC: Firefly luciferase
MAP: Modified atmosphere packaging
MHO: 6-Methyl-5-hepten-2-one
ML: Maximum likelihood
MPTP: Membrane permeability transition pore
NG: Nei-Gojobori
NLP: N-carbamoylputrescine amidase
NOS: Nitric oxide synthase-like
O_2_^•^^−^: Superoxide free radicals
ODC: Ornithine decarboxylase
OE: Overexpressing
OH^•^: Hydroxyl radical
OTC: Ornithine transcarbamylase
REN: Renilla luciferase
ROS: Reactive oxygen species
SOD: Superoxide dismutase
SOH: Sulfenylation
SPDS: Spermidine synthase
SPMS: Spermine synthase
TFs: Transcription factors
WGD: Whole genome duplication
Y1H: Yeast one-hybrid assay
Y2H: Yeast two-hybrid
