# Supplementary figures and images for: PbrWRKY62-PbrADC1 module involves in superficial scald development of Pyrus bretschneideri Rehd.fruit via regulating putrescine biosynthesis
Source: Mol Hortic. 2024 Feb 20;4:6. doi: 10.1186/s43897-024-00081-8 (PMC10877817; doi:10.1186/s43897-024-00081-8)

## Slide 1
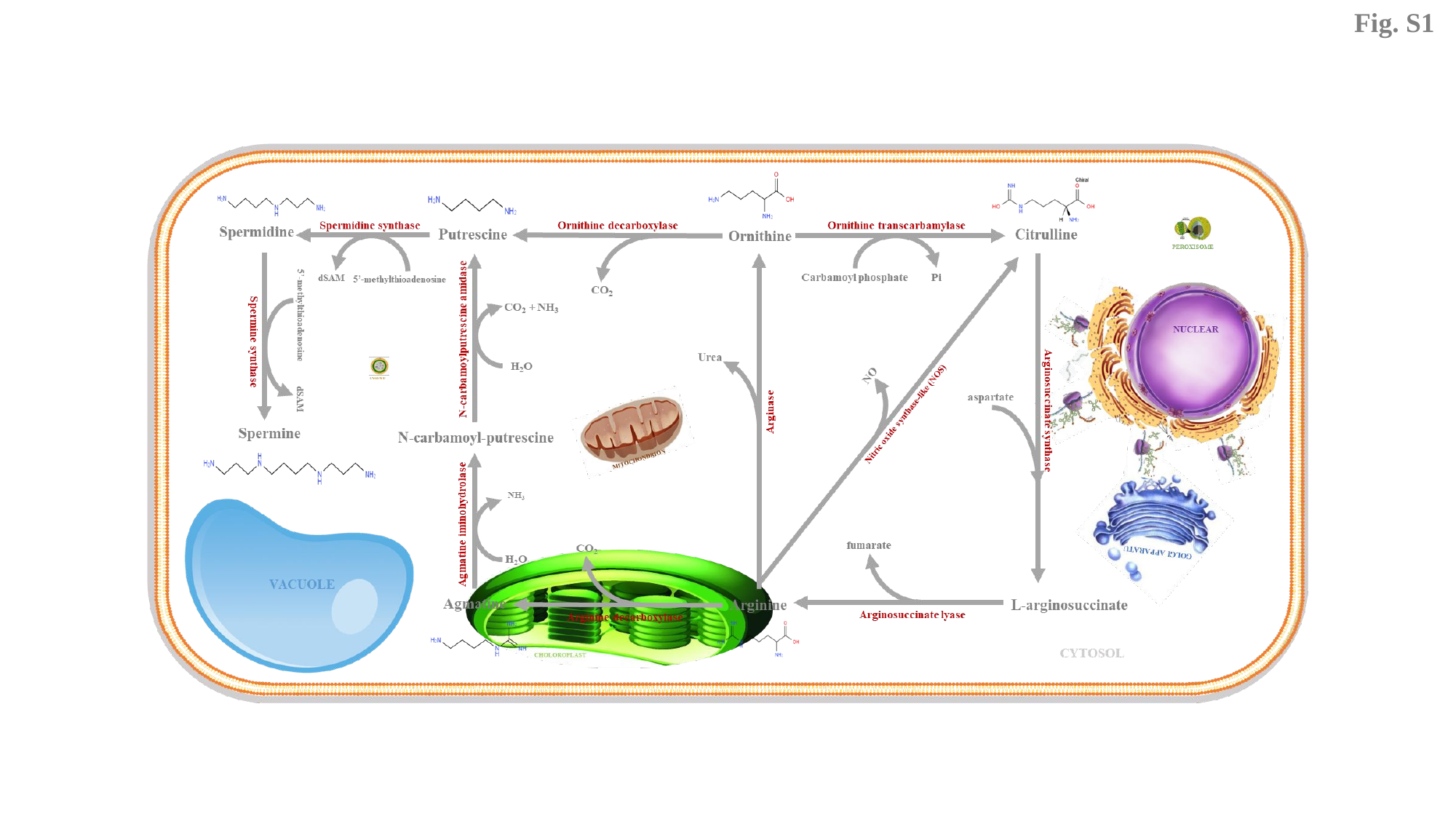

Fig. S1

Supplement: Supplementary file 1 — Additional file 1: Fig. S1. Putrescine metabolic pathway in plants (Kou et al. 2018; Winter et al. 2015). Putrescine in plant could be synthesized either from the decarboxylation of ornithine by ornithine decarboxylase (ODC) or from arginine decarboxylase (ADC) pathway, which is consisted of three enzymes: ADC, agmatine iminohydrolase (AIH), and N-carbamoylputrescine amidase (NLP) (Kou et al. 2018). Upon formation, putrescine could be converted into spermidine and spermine by spermidine synthase (SPDS) and spermine synthase (SPMS) (Winter et al. 2015). On the other hand, ornithine and arginine could be interconverted with the aid of ornithine transcarbamylase (OTC), arginosuccinate synthase (ASS), arginosuccinate lyase (ASL), nitric oxide synthase-like (NOS), and arginase (ARG) (Winter et al. 2015). [file 43897_2024_81_MOESM1_ESM.pptx]

## Slide 1
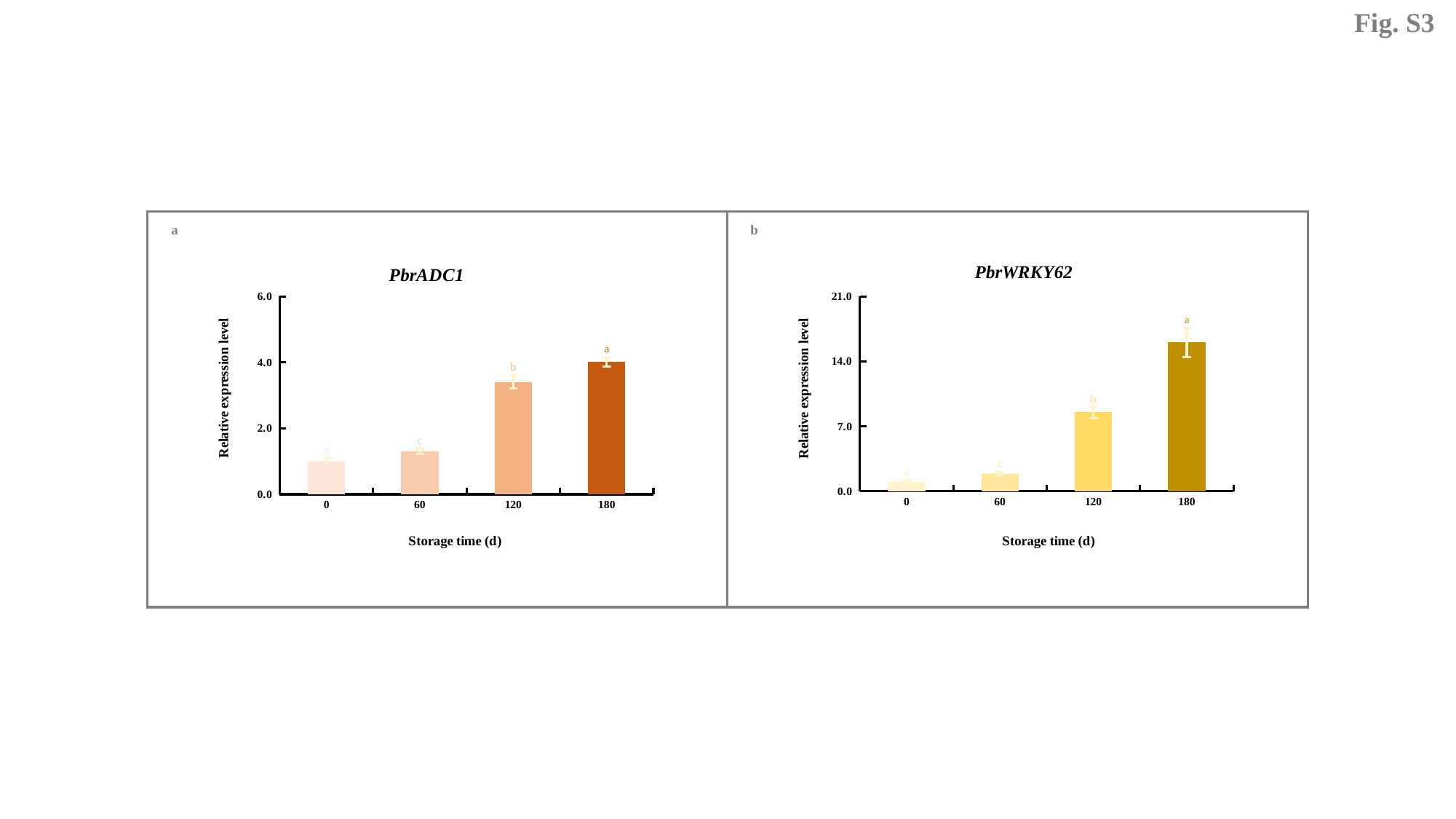

Fig. S3
a
b
### Chart: PbrADC1
| Category | |
|---|---|
| 0 | 1.0 |
| 60 | 1.3066666666666666 |
| 120 | 3.4090000000000003 |
| 180 | 4.008333333333333 |
### Chart: PbrWRKY62
| Category | |
|---|---|
| 0 | 1.0 |
| 60 | 1.8466666666666667 |
| 120 | 8.501 |
| 180 | 16.036666666666665 |

Supplement: Supplementary file 3 — Additional file 3: Fig. S3. RT-qPCR validation of the expression patterns of PbrADC1 (a) and PbrWRKY62 (b). ‘Dangshansuli’ fruit were sampled every 60-d storage at 0.5 ℃ followed by a 7-d shelf life at 25 ℃. Data represented the mean value of three biological replicates. The expression abundances of PbrADC1 and PbrWRKY62 genes in the 0-d fruit were set as 1.0. [file 43897_2024_81_MOESM3_ESM.pptx]

## Slide 1
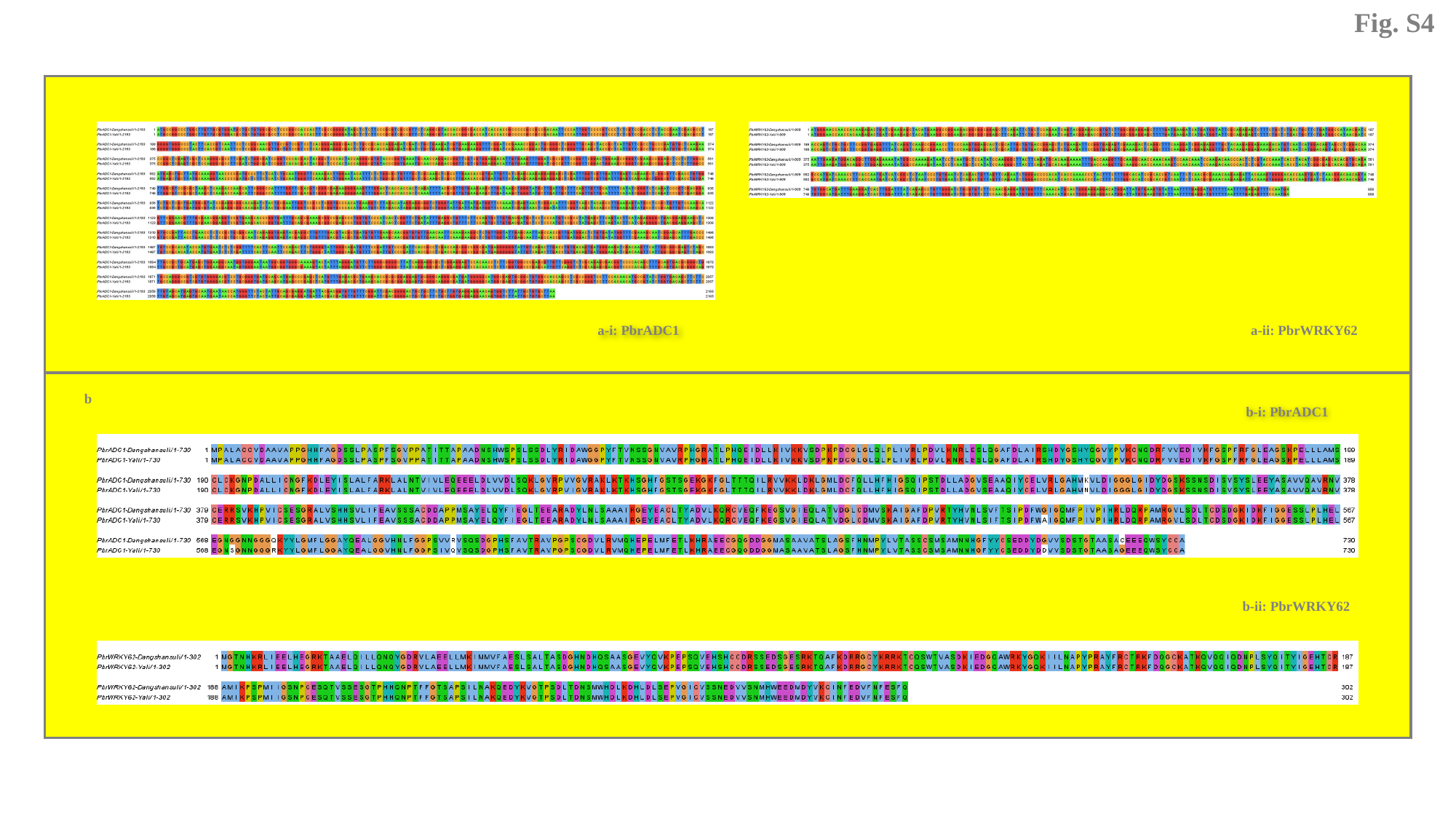

Fig. S4
a
a-ii: PbrWRKY62
a-i: PbrADC1
b
b-i: PbrADC1
b-ii: PbrWRKY62

Supplement: Supplementary file 4 — Additional file 4: Fig. S4. Alignment of CDS sequences and the corresponding protein sequences of PbrADC1 and PbrWRKY62 in ‘Dangshansuli’ and ‘Yali’ fruits. (a) CDS sequences of PbrADC1 (a-i) and PbrWRKY62 (a-ii) genes. (b) Protein sequences of PbrADC1 (b-i) and PbrWRKY62 (b-ii). [file 43897_2024_81_MOESM4_ESM.pptx]

## Slide 1
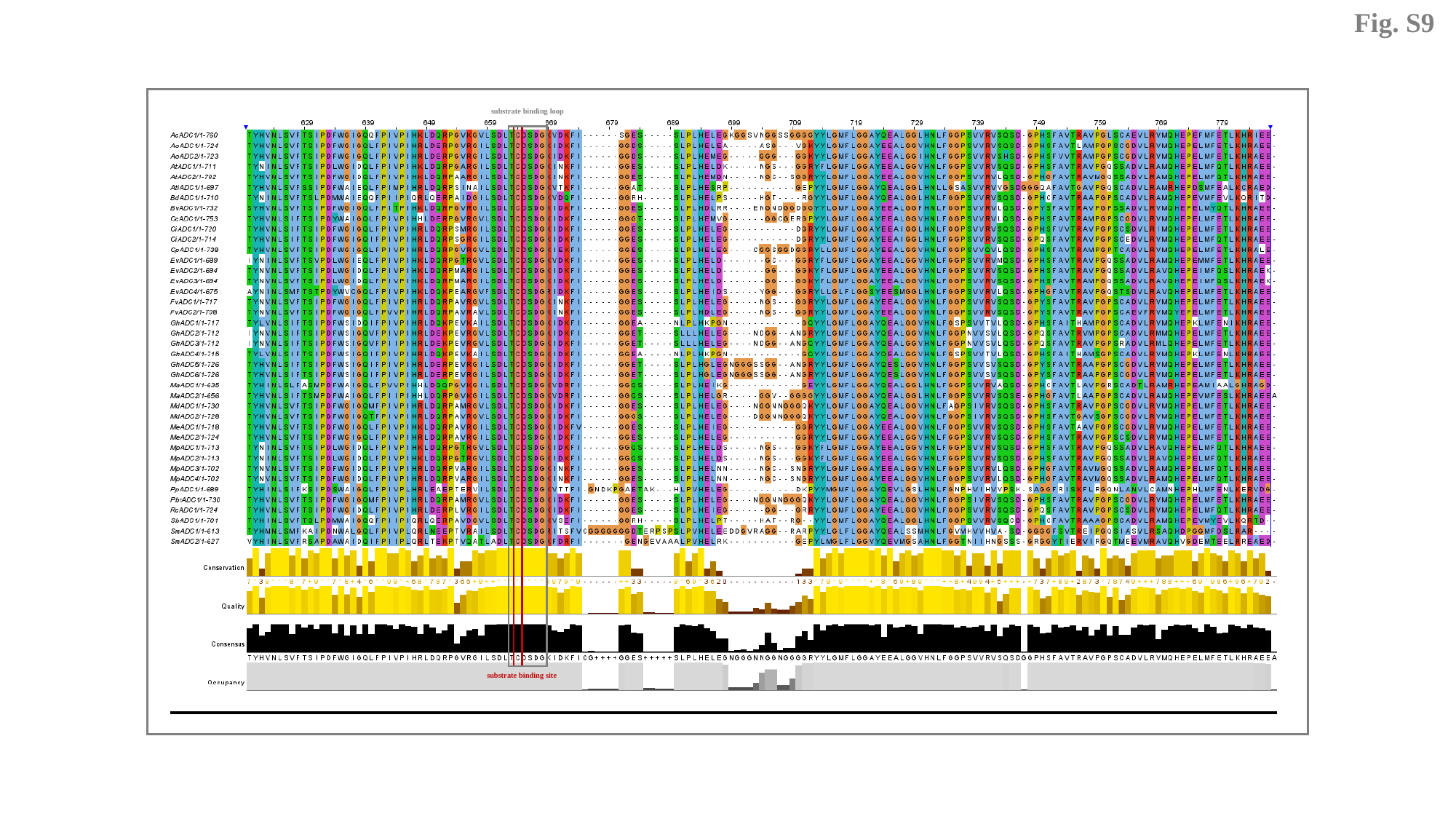

Fig. S9
substrate binding loop
substrate binding site

Supplement: Supplementary file 9 — Additional file 9: Fig. S9. Alignment of plant ADCs by Jalview Version 2. 47 plant ADCs, which were identified from 26 plants, were summarized in Table S7. The substrate-binding residues were highlighted in the red box. Just part of the result was demonstrated. [file 43897_2024_81_MOESM9_ESM.pptx]
