# Supplementary material for: PbrWRKY62-PbrADC1 module involves in superficial scald development of Pyrus bretschneideri Rehd.fruit via regulating putrescine biosynthesis
Source: Mol Hortic. 2024 Feb 20;4:6. doi: 10.1186/s43897-024-00081-8 (PMC10877817; doi:10.1186/s43897-024-00081-8)
Supplement: Supplementary file 2 — Additional file 2: Fig. S2. Information on 21 putrescine-metabolism-related genes in the P. bretschneideri Rehd. genome. (a) Gene localizations. Chromosome numbers were indicated on the inner side, and different colors represented different chromosomes. Additionally, genes that underwent WGD/segmental duplications were connected with red lines. (b) Syntenic relationship of the WGD/segmental duplicated gene pairs. Chromosome/scaffold segments were indicated by grey horizontal lines, and the broad lines with green/blue color represented genes and its transcriptional orientations. Target genes were marked in red color. WGD/segmental duplication gene pairs were connected with bands. (c) Gene structures and the distribution of cis-acting elements. (c-i) Phylogenetic tree. (c-ii) Gene structures. Yellow boxes represent the exons, blue boxes represent the UTRs, while black lines represent the introns. (c-iii) The distribution of cis-acting elements. Boxes with distinct colors represent the different cis-acting elements. 21 putrescine-metabolism-related genes in pear genome were summarized in Table S2. [file 43897_2024_81_MOESM2_ESM.pptx]

## Slide 1
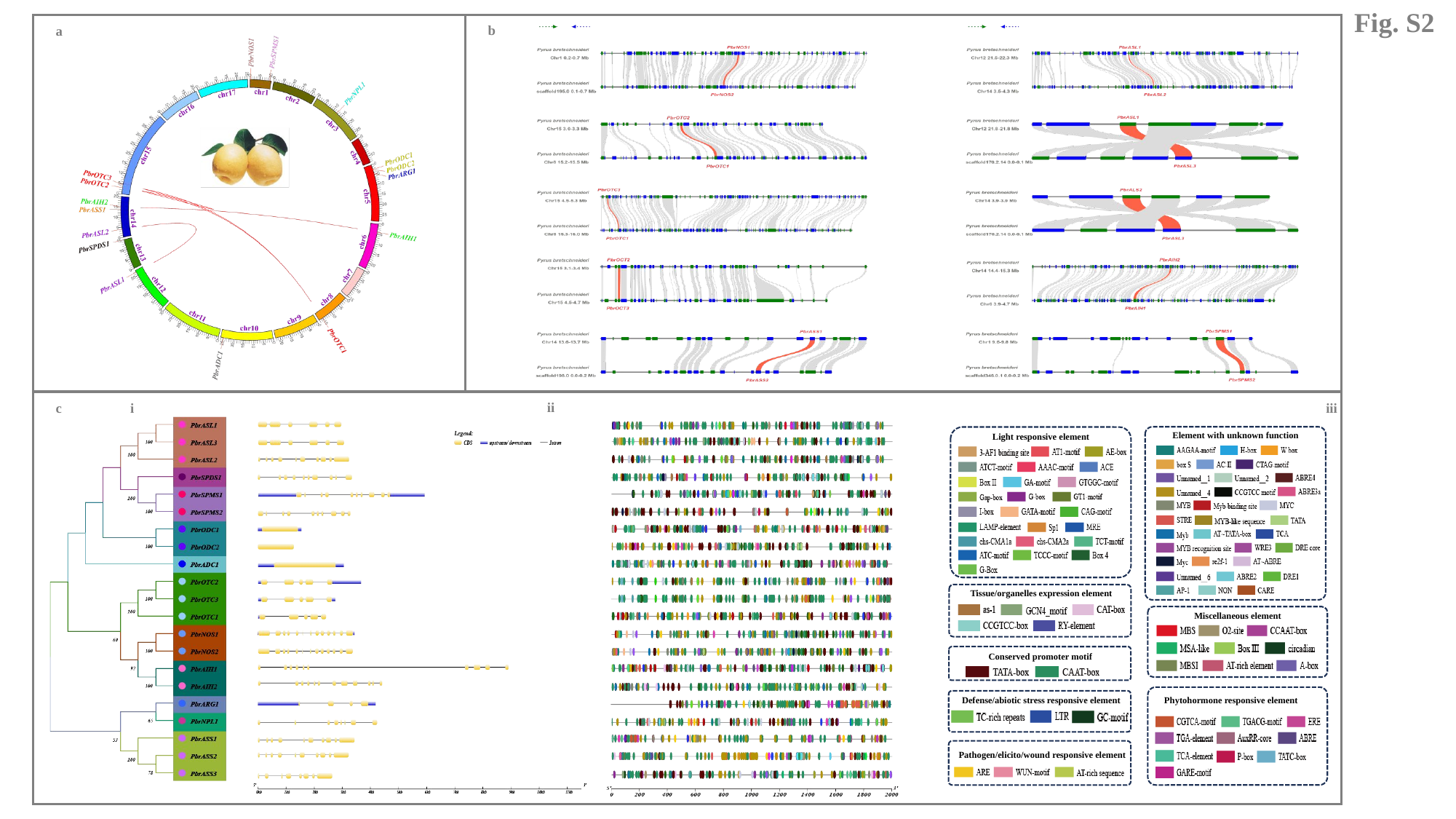

Fig. S2
b
a
ii
c
i
iii
Element with unknown function
Light responsive element
Tissue/organelles expression element
Miscellaneous element
Conserved promoter motif
Phytohormone responsive element
Defense/abiotic stress responsive element
Pathogen/elicito/wound responsive element
