# Supplementary material for: PbrWRKY62-PbrADC1 module involves in superficial scald development of Pyrus bretschneideri Rehd.fruit via regulating putrescine biosynthesis
Source: Mol Hortic. 2024 Feb 20;4:6. doi: 10.1186/s43897-024-00081-8 (PMC10877817; doi:10.1186/s43897-024-00081-8)
Supplement: Supplementary file 5 — Additional file 5: Fig. S5. Transmembrane helix and signal peptide assay of PbrADC1 and PbrWRKY62. (a) Transmembrane helix in PbrADC1 (a-i) and PbrWRKY62 (a-ii). (b) Signal peptide in PbrADC1 (b-i) and PbrWRKY62 (b-ii). [file 43897_2024_81_MOESM5_ESM.pptx]

## Slide 1
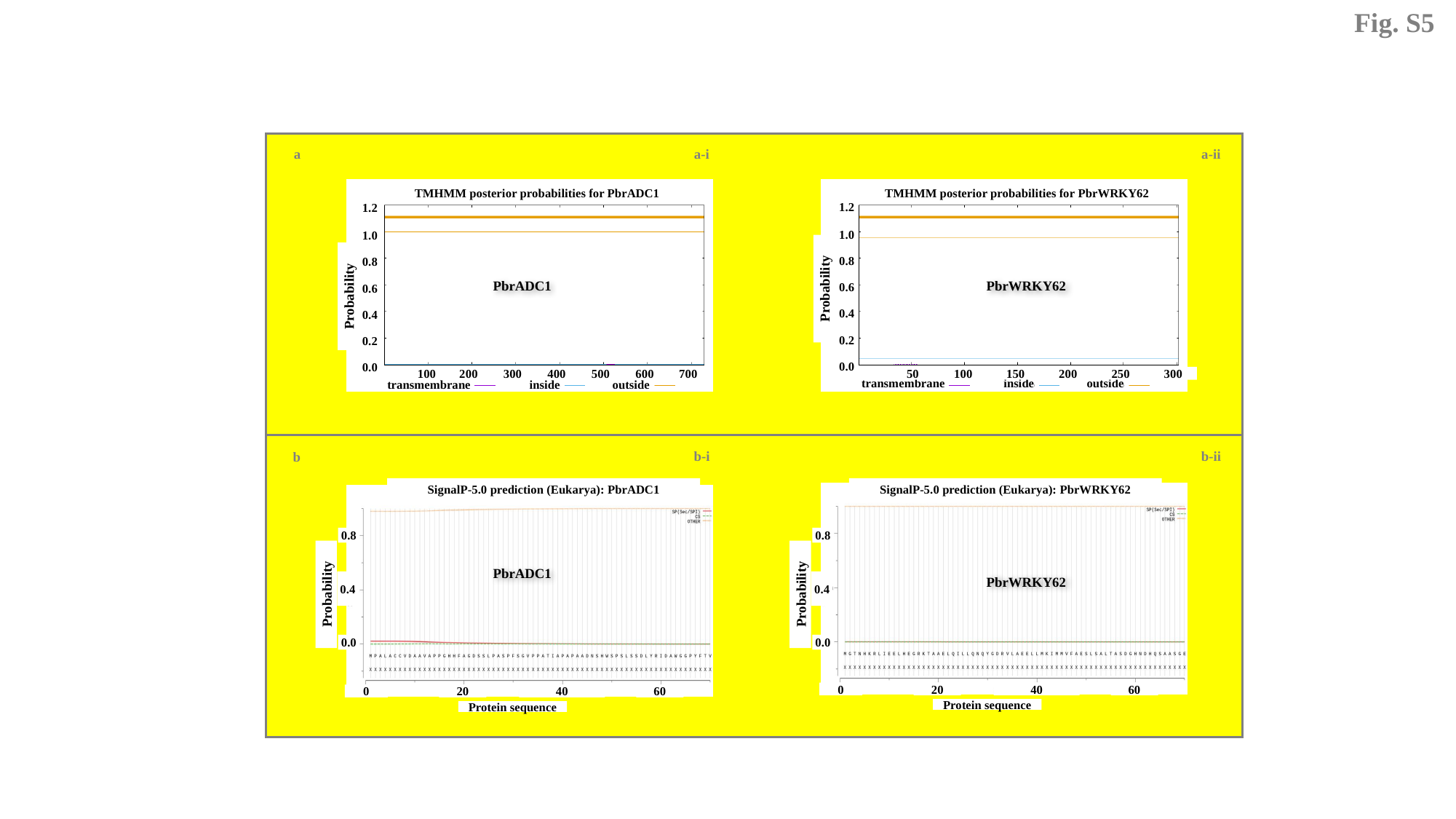

Fig. S5
a
a-ii
a-i
TMHMM posterior probabilities for PbrADC1
TMHMM posterior probabilities for PbrWRKY62
1.2
1.0
0.8
0.6
0.4
0.2
0.0
1.2
1.0
0.8
0.6
0.4
0.2
0.0
PbrADC1
PbrWRKY62
Probability
Probability
50
100
150
200
250
300
100
200
300
400
500
600
700
transmembrane
inside
outside
transmembrane
inside
outside
b-ii
b-i
b
SignalP-5.0 prediction (Eukarya): PbrADC1
SignalP-5.0 prediction (Eukarya): PbrWRKY62
0.8
0.4
0.0
Probability
0.8
0.4
0.0
Probability
PbrADC1
PbrWRKY62
0
20
40
60
Protein sequence
0
20
40
60
Protein sequence
