# Supplementary material for: PbrWRKY62-PbrADC1 module involves in superficial scald development of Pyrus bretschneideri Rehd.fruit via regulating putrescine biosynthesis
Source: Mol Hortic. 2024 Feb 20;4:6. doi: 10.1186/s43897-024-00081-8 (PMC10877817; doi:10.1186/s43897-024-00081-8)
Supplement: Supplementary file 6 — Additional file 6: Fig. S6. Impact of transient genetic transformation of the ripe ‘Yali’ fruit on putrescine abundance. (a) Transient overexpression of PbrADC1 gene. (b) Transient overexpression of PbrWRKY62 gene. The ripe ‘Yali’ fruit transformed with the empty vector was used as a control. The expression abundance of PbrADC1 or PbrWRKY62 in the control fruit was set as 1.0 for RT-qPCR assay. Data represented the mean value of three biological replicates, and different lowercase letters meant significance between samples (p < 0.05). [file 43897_2024_81_MOESM6_ESM.pptx]

## Slide 1
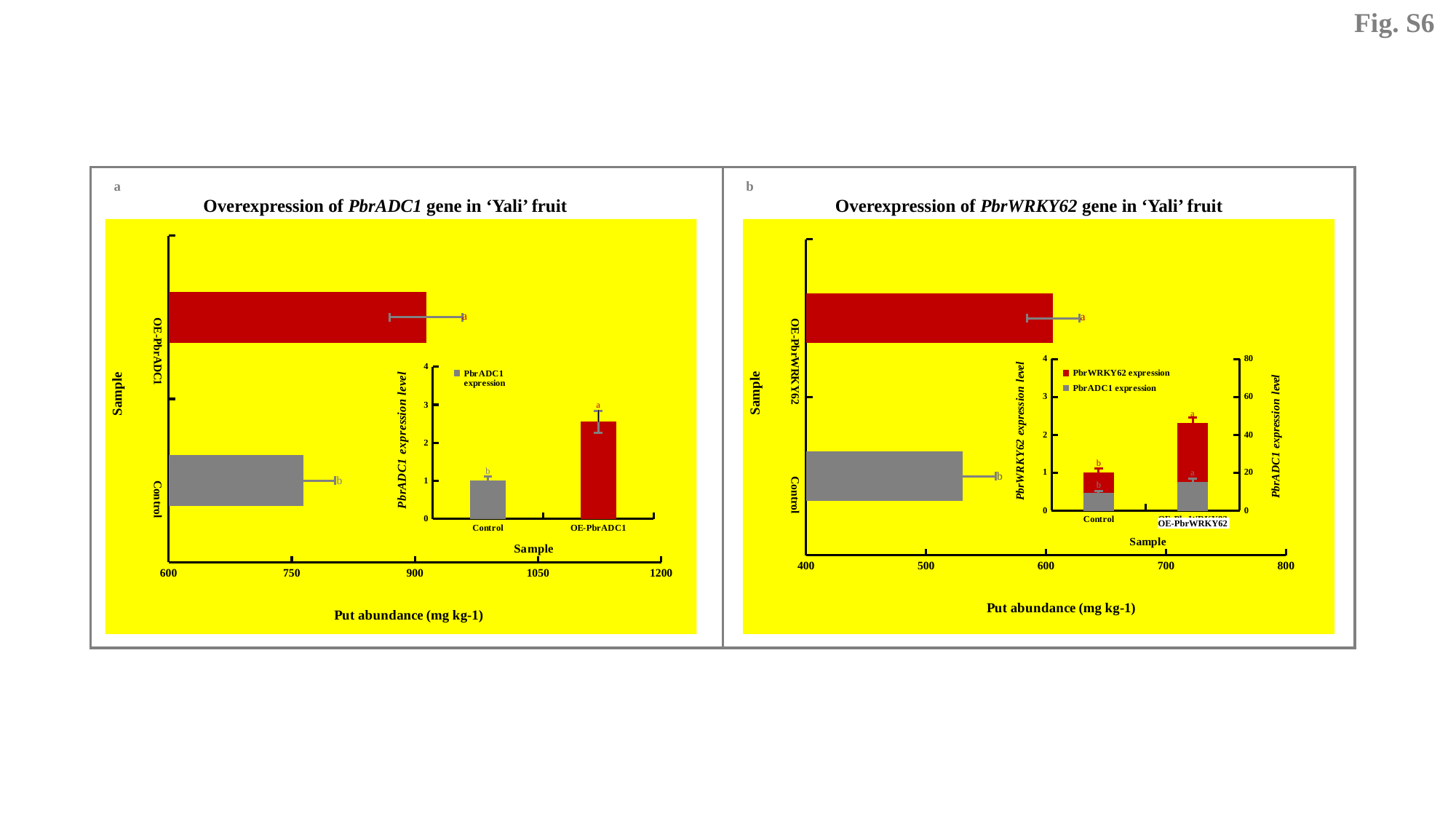

Fig. S6
a
b
Overexpression of PbrADC1 gene in ‘Yali’ fruit
Overexpression of PbrWRKY62 gene in ‘Yali’ fruit
### Chart
| Category | |
|---|---|
| Control | 764.3333333333334 |
| OE-PbrADC1 | 913.6666666666666 |
### Chart
| Category | PbrADC1 expression |
|---|---|
| Control | 1.0 |
| OE-PbrADC1 | 2.548443889 |
### Chart
| Category | Putrescine abundance |
|---|---|
| Control | 530.3333333333334 |
| OE-PbrWRKY62 | 606.0 |
### Chart
| Category | PbrWRKY62 expression | PbrADC1 expression |
|---|---|---|
| Control | 1.0 | 9.236666666666666 |
| OE-PbrWRKY83 | 2.3030000000000004 | 14.843333333333334 |OE-PbrWRKY62
