# Supplementary material for: PbrWRKY62-PbrADC1 module involves in superficial scald development of Pyrus bretschneideri Rehd.fruit via regulating putrescine biosynthesis
Source: Mol Hortic. 2024 Feb 20;4:6. doi: 10.1186/s43897-024-00081-8 (PMC10877817; doi:10.1186/s43897-024-00081-8)
Supplement: Supplementary file 7 — Additional file 7: Fig. S7. Identification of positive transgenic tomato lines at the DNA and RNA levels. (a) PCR assay of the PbrADC1-overexpressing lines (a-i) and PbrWRKY62-overexpressing lines (a-ii) at DNA level. (b) Expression profiles of PbrADC1 (b-i) and PbrWRKY62 (b-ii) genes in tomato fruits. Tomato fruit at 35 DAFB were harvested and then exposed to 4 ℃ for 10 d followed by 20 ℃ storage for 7 d. Data represented the mean value of three biological replicates, and different lowercase letters meant significance between samples (p < 0.05). [file 43897_2024_81_MOESM7_ESM.pptx]

## Slide 1
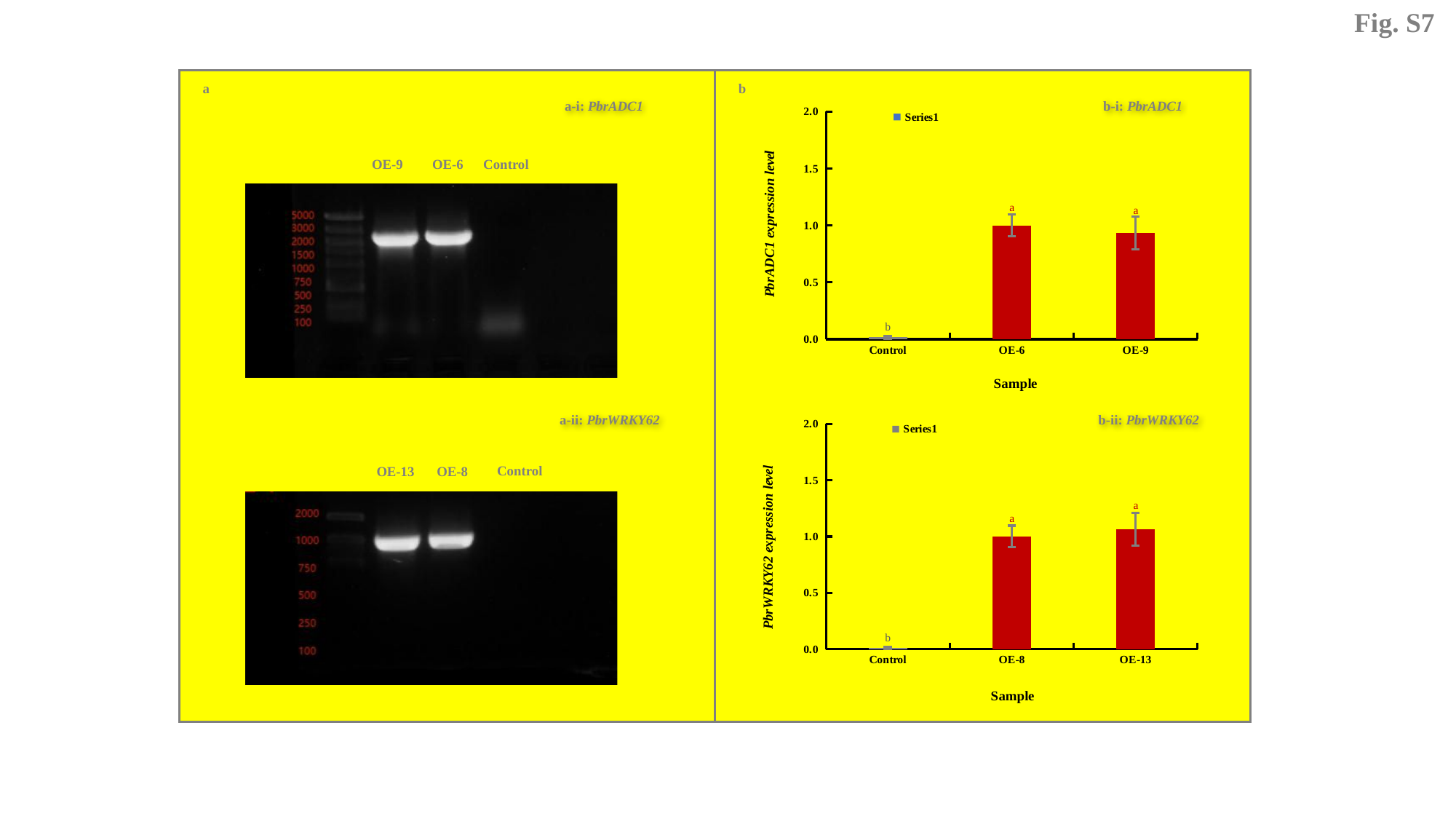

Fig. S7
a
b
a-i: PbrADC1
b-i: PbrADC1
### Chart
| Category | |
|---|---|
| Control | 0.016666666666666666 |
| OE-6 | 1.0 |
| OE-9 | 0.9333333333333332 |Control
OE-9
OE-6
a-ii: PbrWRKY62
b-ii: PbrWRKY62
### Chart
| Category | |
|---|---|
| Control | 0.01 |
| OE-8 | 1.0000000000000002 |
| OE-13 | 1.0633333333333332 |Control
OE-13
OE-8
