# Supplementary material for: PbrWRKY62-PbrADC1 module involves in superficial scald development of Pyrus bretschneideri Rehd.fruit via regulating putrescine biosynthesis
Source: Mol Hortic. 2024 Feb 20;4:6. doi: 10.1186/s43897-024-00081-8 (PMC10877817; doi:10.1186/s43897-024-00081-8)
Supplement: Supplementary file 8 — Additional file 8: Fig. S8. Evolution and characteristics of 47 plant ADCs. (a) Timescale tree of 26 plants drawn by TIMETREE. (b) Gene structures and conserved motifs of 47 plant ADCs. (b-i) Phylogenetic tree. (b-ii) Gene structures. Yellow boxes represent the exons, blue boxes indicate the UTRs, while black lines represent the introns. (b-iii) Conserved motifs. Boxes with distinct colors represent the different motifs. (c) Detailed information on the conserved motifs in plant ADCs. Six conserved motifs were characterized from 47 plant ADCs. Motif 1, 2 & 5 composed the conserved domain ‘Orn_Arg_deC_N’, while Motif 3 & 4 composed the domain ‘d7odca2’. 47 plant ADCs, which were identified from 26 plants, were summarized in Table S7. [file 43897_2024_81_MOESM8_ESM.pptx]

## Slide 1
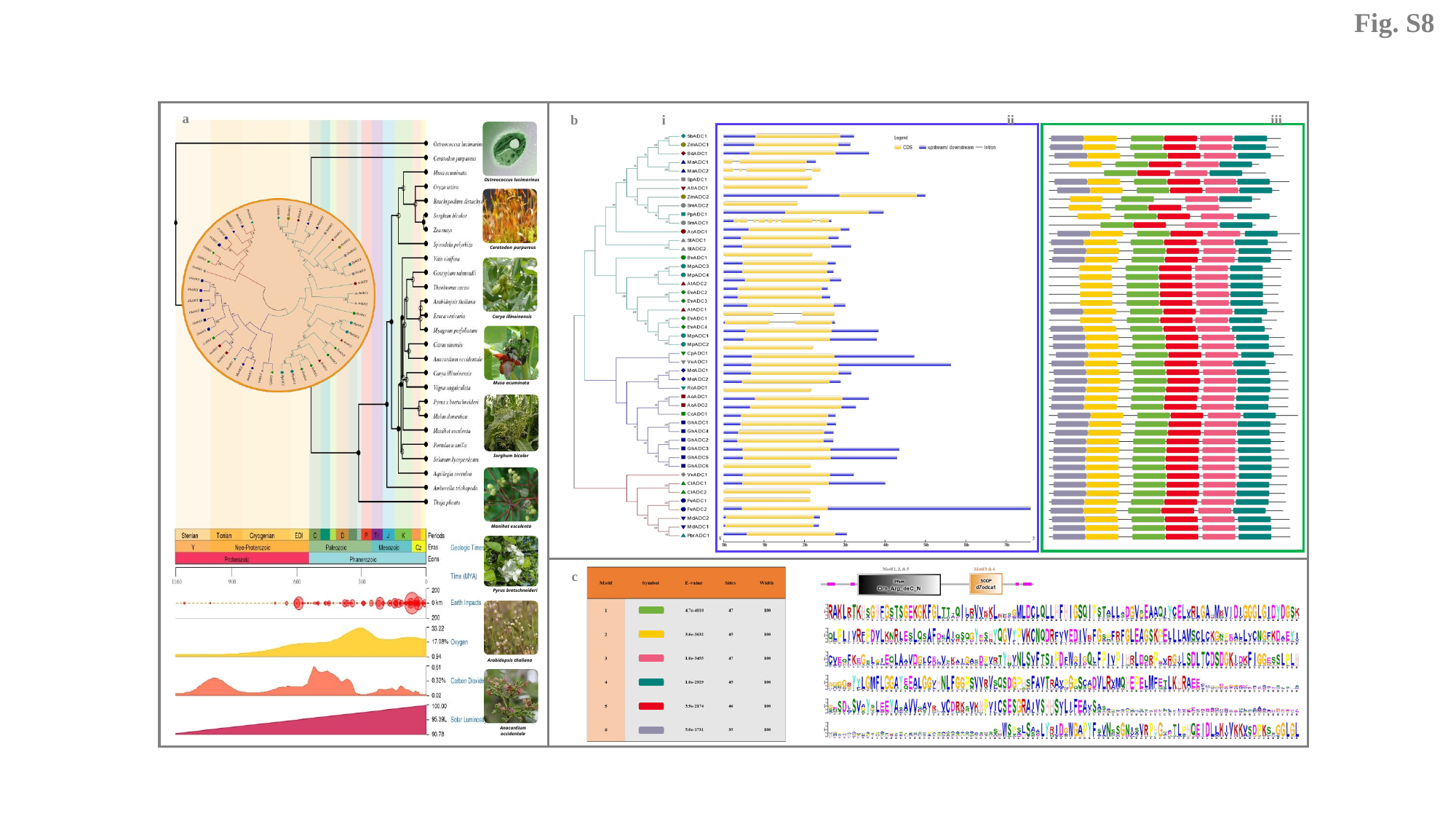

Fig. S8
iii
i
ii
a
b
Ostreococcus lucimarinus
Ceratodon purpureus
 Carya illinoinensis
Musa acuminata
Sorghum bicolor
Manihot esculenta
Pyrus bretschneideri
Arabidopsis thaliana
Anacardium occidentale
c
