# Supplementary material for: PbrWRKY62-PbrADC1 module involves in superficial scald development of Pyrus bretschneideri Rehd.fruit via regulating putrescine biosynthesis
Source: Mol Hortic. 2024 Feb 20;4:6. doi: 10.1186/s43897-024-00081-8 (PMC10877817; doi:10.1186/s43897-024-00081-8)
Supplement: Supplementary file 10 — Additional file 10: Fig. S10. Characterization of transcript factors (TFs) possibly regulating the expression of PbrADC1. (a) Schematic model of the distribution of cis-acting elements in the PbrADC1 promoter. Red, green, and blue ellipses represent the W-box elements, G-box elements, and MYB-binding sites, respectively. (b) Detailed information of cis-acting elements in PbrADC1 promoter. The conserved nucleotide acids were highlighted in red. (c) Expression profiles of TFs and their correlations with PbrADC1 mRNA abundance. (c-i) During the cold storage of ‘Dangshansuli’ fruit. ‘Dangshansuli’ fruit were sampled every 60-d storage at 0.5 ℃ followed by a 7-d shelf life at 25 ℃. Data, adapted from transcriptome assay, represented the mean values of three biological replicates. (c-ii) During ‘Dangshansuli’ fruit development. ‘Dangshansuli’ fruit were sampled at six developmental stages, including fruit-setting stage (15 DAFB), physiological fruit dropping stage (34 DAFB), a month after fruit enlargement stage (81 DAFB), pre-mature stage (110 DAFB), mature stage (145 DAFB), and fruit senescence stage (160 DAFB). Data, adapted from transcriptome assay of the previous study (Zhang et al. 2021), represented the mean value of one biological replicate. (c-iii) Upon superficial scald development in ‘Yali’ fruit. ‘Yali’ fruits, with and without superficial scald, were sampled after -0.5 ℃ for 180 d followed by a 7-d shelf life at 20 ℃.Data, adapted from transcriptome assay, represented the mean values of two biological replicates.PbrWRKYs, PbrbZIPs, and PbrMYBs genes were characterized from the P. bretschneideri Rehd. genome (Cao et al. 2016; Huang et al. 2015; Ma et al. 2021). Color scale represents normalized log2-transformed (FPKM + 1), where red indicates a high level, blue represents a low level, and white indicates a medium level. Absolute correlation coefficients between PbrADC1 and TFs ≥ 0.8 were visualized in the heatmap, where red lines demonstrated extremely strong [file 43897_2024_81_MOESM10_ESM.pptx]

## Slide 1
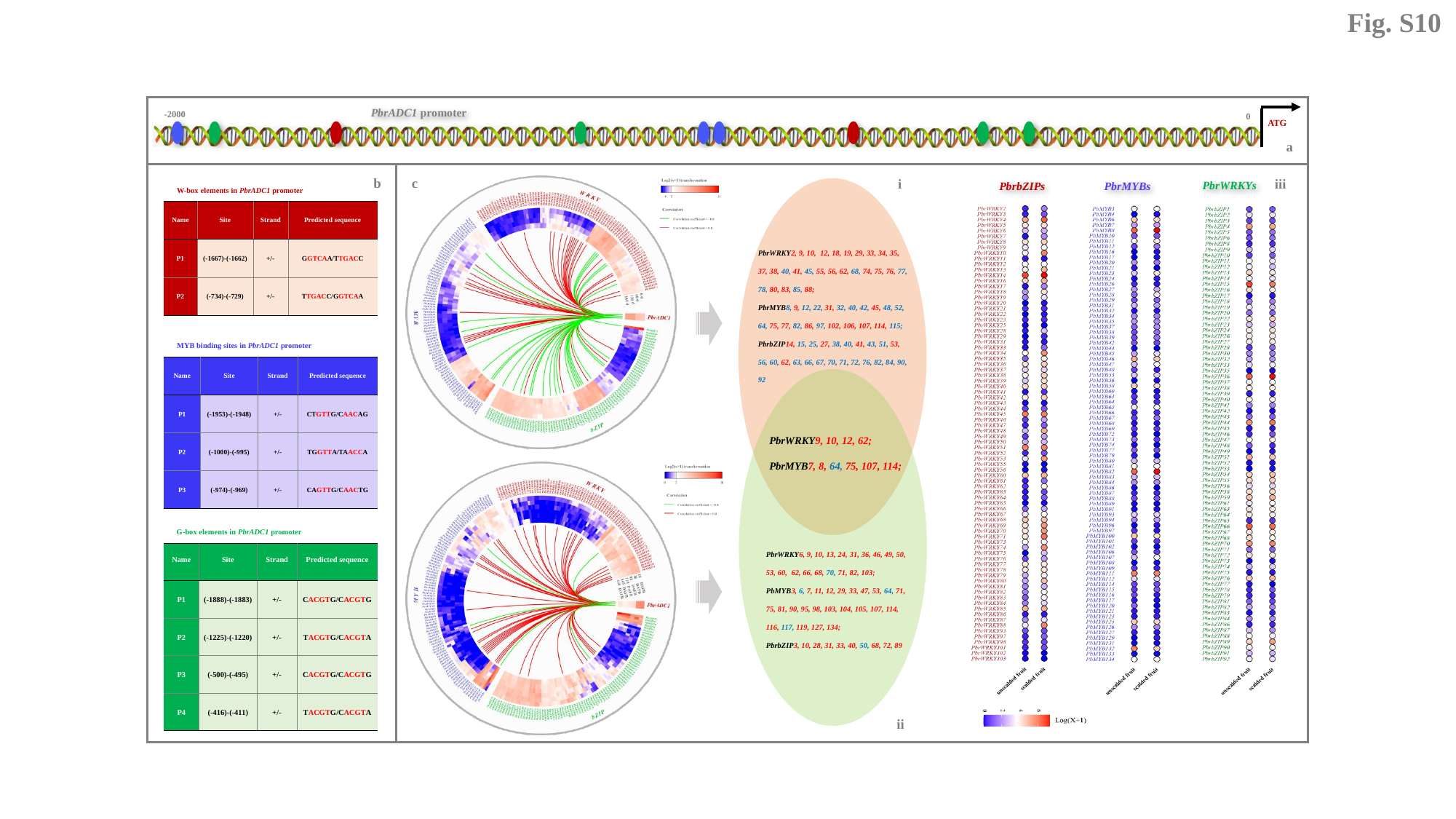

Fig. S10
PbrADC1 promoter
-2000
0
ATG
a
b
c
i
iii
PbrWRKYs
PbrbZIPs
PbrMYBs
PbrWRKY2, 9, 10, 12, 18, 19, 29, 33, 34, 35, 37, 38, 40, 41, 45, 55, 56, 62, 68, 74, 75, 76, 77, 78, 80, 83, 85, 88;
PbrMYB8, 9, 12, 22, 31, 32, 40, 42, 45, 48, 52, 64, 75, 77, 82, 86, 97, 102, 106, 107, 114, 115;
PbrbZIP14, 15, 25, 27, 38, 40, 41, 43, 51, 53, 56, 60, 62, 63, 66, 67, 70, 71, 72, 76, 82, 84, 90, 92
PbrWRKY9, 10, 12, 62;
PbrMYB7, 8, 64, 75, 107, 114;
PbrWRKY6, 9, 10, 13, 24, 31, 36, 46, 49, 50, 53, 60, 62, 66, 68, 70, 71, 82, 103;
PbMYB3, 6, 7, 11, 12, 29, 33, 47, 53, 64, 71, 75, 81, 90, 95, 98, 103, 104, 105, 107, 114, 116, 117, 119, 127, 134;
PbrbZIP3, 10, 28, 31, 33, 40, 50, 68, 72, 89
W-box elements in PbrADC1 promoter
| Name | Site | Strand | Predicted sequence |
| --- | --- | --- | --- |
| P1 | (-1667)-(-1662) | +/- | GGTCAA/TTGACC |
| P2 | (-734)-(-729) | +/- | TTGACC/GGTCAA |
MYB binding sites in PbrADC1 promoter
| Name | Site | Strand | Predicted sequence |
| --- | --- | --- | --- |
| P1 | (-1953)-(-1948) | +/- | CTGTTG/CAACAG |
| P2 | (-1000)-(-995) | +/- | TGGTTA/TAACCA |
| P3 | (-974)-(-969) | +/- | CAGTTG/CAACTG |
G-box elements in PbrADC1 promoter
| Name | Site | Strand | Predicted sequence |
| --- | --- | --- | --- |
| P1 | (-1888)-(-1883) | +/- | CACGTG/CACGTG |
| P2 | (-1225)-(-1220) | +/- | TACGTG/CACGTA |
| P3 | (-500)-(-495) | +/- | CACGTG/CACGTG |
| P4 | (-416)-(-411) | +/- | TACGTG/CACGTA |
ii
